# Ventral tegmental area GABA neurons mediate stress-induced blunted reward-seeking in mice

**DOI:** 10.1038/s41467-021-23906-2

**Published:** 2021-06-10

**Authors:** Daniel C. Lowes, Linda A. Chamberlin, Lisa N. Kretsge, Emma S. Holt, Atheir I. Abbas, Alan J. Park, Lyubov Yusufova, Zachary H. Bretton, Ayesha Firdous, Armen G. Enikolopov, Joshua A. Gordon, Alexander Z. Harris

**Affiliations:** 1 Department of Psychiatry, Columbia University, College of Physicians and Surgeons, New York, NY USA; 2 VA Portland Health Care System, Department of Behavioral Neuroscience and Department of Psychiatry, Oregon Health & Science University, Portland, OR USA; 3 Division of Systems Neuroscience, New York State Psychiatric Institute, New York, NY USA; 4 The Mortimer B. Zuckerman Mind Brain Behavior Institute at Columbia University, New York, NY USA; 5 Department of Neuroscience and Kavli Institute for Brain Science, Columbia University Medical Center, New York, NY USA; 6 National Institute of Mental Health, Bethesda, MD USA

**Keywords:** Striatum, Neural circuits, Reward, Stress and resilience, Neurophysiology

## Abstract

Decreased pleasure-seeking (anhedonia) forms a core symptom of depression. Stressful experiences precipitate depression and disrupt reward-seeking, but it remains unclear how stress causes anhedonia. We recorded simultaneous neural activity across limbic brain areas as mice underwent stress and discovered a stress-induced 4 Hz oscillation in the nucleus accumbens (NAc) that predicts the degree of subsequent blunted reward-seeking. Surprisingly, while previous studies on blunted reward-seeking focused on dopamine (DA) transmission from the ventral tegmental area (VTA) to the NAc, we found that VTA GABA, but not DA, neurons mediate stress-induced blunted reward-seeking. Inhibiting VTA GABA neurons disrupts stress-induced NAc oscillations and rescues reward-seeking. By contrast, mimicking this signature of stress by stimulating NAc-projecting VTA GABA neurons at 4 Hz reproduces both oscillations and blunted reward-seeking. Finally, we find that stress disrupts VTA GABA, but not DA, neural encoding of reward anticipation. Thus, stress elicits VTA-NAc GABAergic activity that induces VTA GABA mediated blunted reward-seeking.

## Abstract (web summary)

Acute stress transiently disrupts reward-seeking behaviour and repeated stress exposure produces lasting anhedonia-like behaviour in rodents. Here, the authors show that stress triggers GABAergic activity in the ventral tegmental area which blunts reward-seeking behaviour in mice.

## Introduction

In humans and rodents, acute stress transiently disrupts reward-seeking^1,2^, and repeated stress exposure produces lasting reward-seeking deficits^3,4^. Dopamine (DA) transmission between the ventral tegmental area (VTA) and the nucleus accumbens (NAc) lie at the core of reward processing^5–7^. Yet, despite 40 years of research into the DA anhedonia hypothesis^8^, we do not fully understand how stress disrupts reward processing and its underlying neural circuitry. Past studies investigating VTA-NAc DA transmission in stress-induced blunted reward-seeking have yielded conflicting results^9,10^. Crucially, these studies relied on indirect measures of reward circuit activity, such as in vitro or baseline in vivo recordings of DA neural firing rates. Here, we record reward circuit activity during both stress and subsequent reward-seeking to directly determine that VTA GABA activity links acute stress and blunted reward-seeking.

## Results

### Restraint stress induces low-frequency NAc LFP oscillations and impairs reward anticipation

**Fig. 1:**
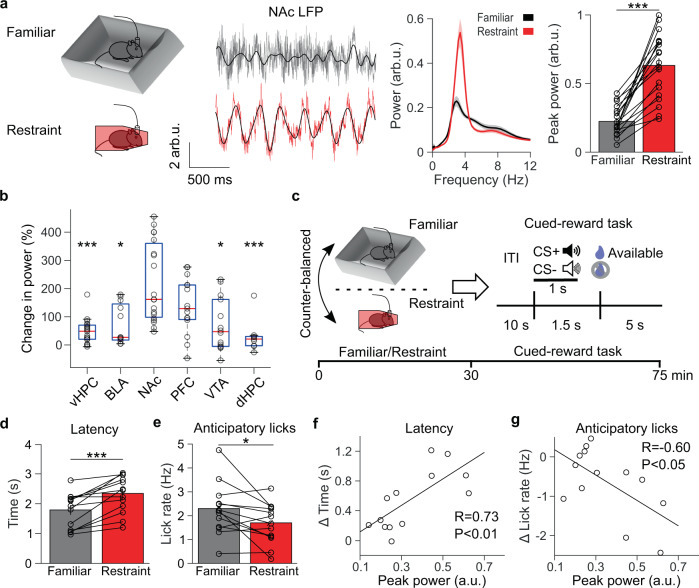
Restraint stress re-organizes the NAc LFP and impairs subsequent reward-seeking. **a** Far left: experimental design. Left: representative traces of the nucleus accumbens (NAc) local field potential (LFP) of a mouse freely exploring a familiar environment (gray) or during restraint (red), overlaid with the 2–7 Hz filtered signal (black). Voltage is normalized to the root mean square of a baseline recording and reported in arbitrary units (arb.u.). Right: average NAc LFP power spectra of mice exploring a familiar environment (black) or during restraint stress (red). Far right: restraint increases peak NAc power in the 4 (2–7) Hz range (***P = 0.00013 two-tailed signed-rank test, *n* = 19 mice). Data are mean ± s.e.m. **b** The percent change in peak 4 Hz power from familiar to restraint was larger in the NAc than the ventral hippocampus (vHPC), basolateral amygdala (BLA), VTA, and dorsal hippocampus (dHPC) (*P* < 0.0001 Kruskal–Wallis test; post hoc Tukey–Kramer comparisons ***P_vHPC_ < 0.0001, *n*_vHPC_ = 19 mice, *P_BLA_ = 0.014, *n*_BLA_ = 12 mice, *P*_PFC_ = 0.20, *n*_PFC_ = 15 mice, *P_VTA_ = 0.015 *n*_VTA_ = 14 mice, ***P_dHPC_ < 0.0001, *n*_dHPC_ = 9 mice, *n*_NAc_ = 10 mice). Data are plotted with median, upper and lower quartiles, and 1.5x interquartile range. **c** Experimental design. **d** Restraint stress increases the time to first lick during the reward availability period in the cued-reward task (***P = 0.00049 two-tailed signed-rank test, *n* = 13 mice). Data are mean ± s.e.m. **e** Restraint stress decreases average anticipatory lick rate between CS+ onset and reward availability (*P = 0.011 two-tailed paired *t* test, *n* = 13 mice). Data are mean ± s.e.m. **f** Relationship between peak 4 Hz NAc power during restraint and the change in latency to reward retrieval from after familiar environment exploration to after restraint (**P < 0.0049 Pearson correlation, *n* = 13 mice) **g** The same as **f**, but for the change in anticipatory lick rate from after familiar environment exploration to after restraint (*P = 0.029 Pearson correlation, *n* = 13 mice).

To screen for the stress-induced neural activity that causes blunted reward-seeking, we recorded local field potentials (LFP)—which reflect local synchronous synaptic activity^11,12^—across brain areas implicated in depression, including the prefrontal cortex^13,14^, NAc^4,13^, dorsal and ventral hippocampus^13,15^, basolateral amygdala^13^, and VTA^4,13^, as mice either explored a familiar environment or underwent 30 mins of acute restraint stress. A prominent low-frequency (2–7 Hz, henceforth 4 Hz) oscillation of LFP activity emerged in the NAc during restraint stress (Fig. 1a and Supplementary Fig. 1a). A restraint-induced oscillation also appeared in other brain regions, including the prefrontal cortex, where stress has previously been reported to induce low-frequency oscillations (Fig. 1b)^16^. However, simultaneous recordings revealed that the restraint-induced oscillation was largest in the NAc (Supplementary Fig. 1b). The magnitude of the restraint-induced oscillation did not differ between the core and shell of the NAc (Supplementary Fig. 1c). This restraint-induced oscillation straddles the hippocampal theta frequency range (4–12 Hz), yet simultaneous recordings in the hippocampus and NAc revealed that the restraint-induced oscillation was distinct from hippocampal theta (Supplementary Fig. 1d). We did not observe similar oscillations during periods of voluntary immobility in the familiar environment, suggesting that this neural activity reflects restraint, rather than decreased movement (Supplementary Fig. 1e). As further evidence that this oscillation is a signature of acute stress, it persisted at the same magnitude throughout the restraint session and only abated when the mice were released from restraint (Supplementary Fig. 2a). Restrained mice spent ~5% of their time struggling, and periods of struggling were associated with decreased 4 Hz power (Supplementary Fig. 2b). Moreover, exposure to another stressor (tail suspension) induced a similar magnitude low-frequency oscillation in the NAc that decreased during struggling events (Supplementary Fig. 2c), confirming that passive stressors elicit a 4 Hz NAc oscillation. Although recent work has proposed that respiration-driven oscillations originating in the olfactory bulbs and piriform cortex represent the true source of NAc oscillations^17,18^, we found only modest coherence between the NAc LFP and respirations, which was not increased by restraint (Supplementary Fig. 2d, e). Thus, rhythmic low-frequency NAc LFP activity appears to be a neural signature of acute stress.

We wondered if this stress-induced NAc activity could cause blunted reward-seeking. To test this hypothesis, we trained mice to collect rewards after hearing a reward-predicting cue (CS+). After a 1.5 s delay from CS+ onset, a reward was available for 5 s. Once the mice reached stable performance, we recorded VTA-NAc reward circuit activity from them over 2 days as they either explored a familiar environment or underwent acute restraint stress in a counterbalanced order (Fig. 1c).

Immediately after undergoing 30 mins of restraint stress, freely moving mice showed increased latency to retrieve rewards and decreased anticipatory (delay period) licking relative to mice exposed to a neutral environment for an equivalent period (Fig. 1d, e). This decreased reward anticipation returned to control levels when measured the following day (Supplementary Fig. 2f). Stress did not significantly decrease general mouse movement (Supplementary Fig. 2g) or non-reward-predicting cue (CS−) associated behavior (Supplementary Fig. 2h), suggesting a selective deficit in reward anticipation. We performed experiments in both male and female mice but found no significant sex*stress interactions (Supplementary Fig. 8). Notably, the magnitude of stress-induced oscillations correlated with the degree of reward-seeking impairment, suggesting that 4 Hz NAc oscillations reflect the extent to which stress induces circuit plasticity which disrupts subsequent reward-seeking (Fig. 1f, g).

**Fig. 2:**
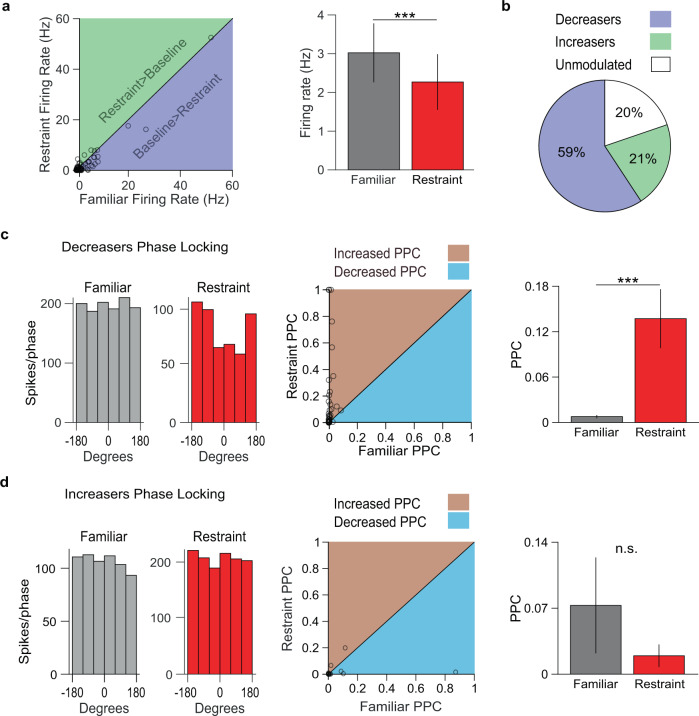
Restraint-inhibited NAc units phase-lock to local low-frequency oscillations. **a** Left: relationship between NAc firing rates in familiar environment and restraint, with line of equality for reference. Right: summary of data from left panel. Restraint inhibited overall NAc neuron firing rate (***P < 0.0001 two-tailed signed-rank tests, *n* = 81 neurons). Data are mean ± s.e.m. **b** Distribution of NAc neurons excited, inhibited, and unmodulated by restraint. **c** Left: the spike-phase relationship for example restraint-inhibited NAc neuron. The activity was uniformly distributed across 4 Hz filtered NAc LFP phase angles during familiar environment exploration, but phase-locked during restraint. Center: Relationship between the pairwise phase consistency (PPC) of restraint-inhibited NAc neurons during familiar environment exploration and restraint, with line of equality for reference. Right: summary of data from center panel. Restraint-inhibited NAc neurons increased phase-locking to the 4 Hz filtered NAc LFP during restraint (***P < 0.0001, two-tailed signed-rank test, *n* = 48 neurons). Data are mean ± s.e.m. **d** Same as **c**, but with restraint-excited NAc neurons. Restraint-excited NAc neurons did not increase phase locking to the NAc LFP. (*P* = 0.33, two-tailed signed-rank test, *n* = 17 neurons). Data are mean ± s.e.m.

Since low-frequency oscillations reflect synchronous subthreshold synaptic inputs to a structure^11^, we expected stress-induced oscillations to modulate NAc neural firing rates. Indeed, restraint stress robustly affected NAc single-unit activity. The firing rates of ~80% of NAc single units were significantly modulated by restraint. Most significantly modulated neurons had reduced activity, while a minority had increased firing rates (Fig. 2a, b). On average, NAc single-unit firing rates became rhythmically entrained to the restraint-induced NAc oscillation (Supplementary Fig. 3a, b). As we observed a variable range of firing rates in the recorded NAc units, we clustered NAc units into putative subtypes to determine the possible effect of restraint on different neuronal subpopulations. We classified NAc units as putative medium spiny neurons (pMSNs), putative tonically active neurons (pTANs), or putative fast-spiking interneurons (pFSIs) based on waveform valley-amplitude ratio, interspike interval coefficient of variation (ISI CV), and baseline firing rate (Supplementary Fig. 3c)^19–21^. Of these clusters, only pMSNs exhibited increased phase-locking to 4 Hz NAc oscillations during restraint, although we identified relatively few pTANs and pFSIs, commensurate with their sparse prevalence in the NAc^22^ (Supplementary Fig. 3d–f). In contrast to NAc single units, prefrontal cortex single units did not phase-lock to the oscillation recorded in the prefrontal cortex (Supplementary Fig. 3g), suggesting that the oscillation originates in the NAc and may be volume conducted to other brain regions. Interestingly, although NAc single units with significantly decreased firing rates showed an increase in phase locking, those with significantly increased firing rates did not, suggesting that the stress-induced oscillation reflects a net inhibitory input to the NAc (Fig. 2c, d).

### VTA neural activity is necessary for the NAc restraint oscillation

**Fig. 3:**
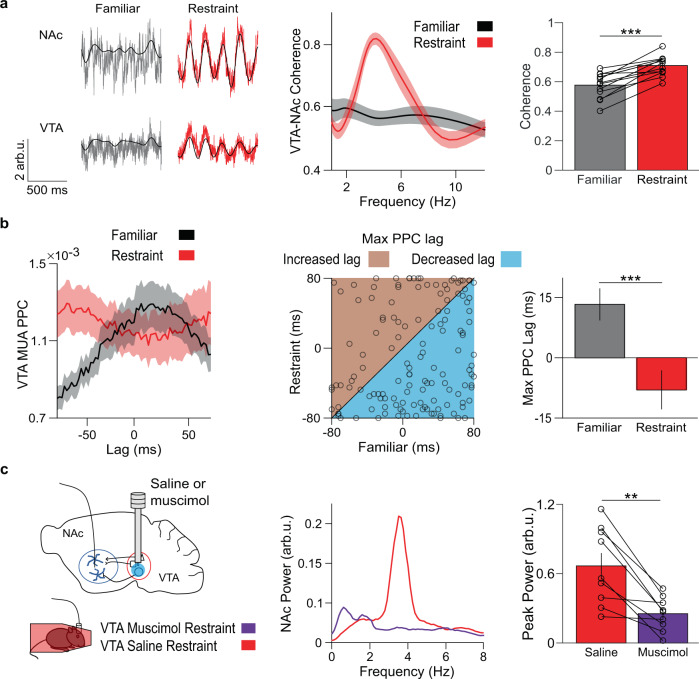
VTA neural activity leads NAc activity during restraint. **a** Left: example traces of NAc and VTA LFPs during familiar environment exploration (gray) or restraint (red), overlaid with the 4 Hz filtered signal (black). Center: coherence of VTA and NAc LFPs. Right: restraint increases average VTA-NAc coherence in the 4 Hz range (***P = 0.00024 two-tailed paired *t* test, *n* = 13 mice). Data are mean ± s.e.m. **b** Left: PPC between VTA MUA and the 4 Hz filtered NAc LFP at different MUA time lags. Restraint increased synchrony of NAc 4 Hz phase with past VTA MUA. Center: relationship between lag of max VTA MUA-NAc PPC during familiar environment exploration and restraint, with the line of equality for reference. Right: summary of data from the center panel. Restraint reorganized VTA MUA from predominantly lagging NAc phase to predominantly leading NAc phase (***P = 0.00033 two-tailed signed-rank test, *n* = 138 multi-units). Data are mean ± s.e.m. **C** Left: experimental design. Center: example NAc LFP power spectra following VTA infusion of saline (red) or muscimol (violet). Right: Muscimol reduced maximum 4 Hz NAc power during restraint (**P = 0.0020 two-tailed signed-rank test, *n* = 9 mice). Data are mean ± s.e.m.

We hypothesized that since the VTA projects densely to the NAc and has a key role in reward processing, it could be the source of stress-induced oscillations. Consistent with this hypothesis, coherence between VTA and NAc LFPs rose sharply during restraint stress in the same frequency range as the restraint-induced oscillation (Fig. 3a). This increase in VTA-NAc coherence was significantly greater than any changes in NAc coherence with the prefrontal cortex, amygdala, or hippocampus (Supplementary Fig. 3h). Since the VTA and NAc reciprocally communicate, we inferred directionality by examining the lag at which phase-locking peaks between VTA multiunit activity (MUA) and the NAc restraint-induced oscillation^23^. In the familiar environment, VTA MUA was best phase-locked to the NAc LFP of the past. By contrast, during restraint stress, VTA MUA was best phase-locked to the NAc LFP of the future. These results suggest that during restraint stress the predominant directionality of reward circuit activity is from the VTA to the NAc (Fig. 3b).

We next examined the role of VTA inputs in driving the restraint-induced NAc oscillation. We infused the GABA_A_ agonist muscimol (0.5 µg/side) or saline control into the VTA while recording NAc LFP activity in restrained mice. Muscimol treatment significantly reduced the restraint-induced oscillation, suggesting that VTA activity is necessary for restraint-induced NAc activity (Fig. 3c).

### VTA DA activity during restraint does not induce stress-induced NAc oscillations

**Fig. 4:**
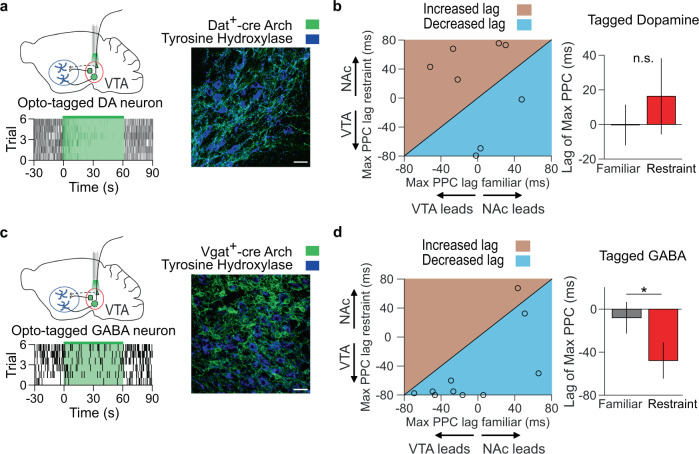
VTA GABA activity leads to low-frequency NAc oscillations during restraint. **a** Top left: experimental design. Bottom left: raster plot of opto-tagged DA neuron. Right: immunofluorescent image of archaerhodopsin (Arch) expression (green) in a Dat-ires-Cre mouse VTA, with tyrosine hydroxylase counterstain (blue). Scale bar is 30 µm. Representative of 6 experiments. **b** Left: Relationship between lag of max VTA DA-NAc PPC during familiar environment exploration and restraint, with line of equality for reference. Right: Summary of data from left panel. Restraint did not change the phase-locking direction of the NAc LFP with VTA DA neurons (*P* = 0.54, two-tailed paired *t* test, *n* = 8 neurons). Data are mean ± s.e.m. **c** Example raster plot and immunofluorescent image from a Vgat-ires-Cre mouse. Scale bar is 30 µm. Representative of six experiments. **d** The same as **b**, but for opto-tagged GABA neurons in a Vgat-ires-Cre mouse. Restraint shifted 4 Hz NAc LFP phase-locking toward past VTA GABA neuron activity (*P = 0.012 two-tailed signed-rank test, *n* = 10 neurons). Data are mean ± s.e.m.

Given their role in reward processing, VTA DA neurons are a prime candidate for mediating anhedonia^8^. To determine whether these neurons provide input to the NAc during restraint stress, we injected Cre-dependent adeno-associated virus (AAV) carrying archaerhodopsin (Arch) into the VTA of Dat-ires-Cre mice to optogenetically tag DA neurons, identified by comparing firing rates between light ON and light OFF epochs with bootstrapping (Fig. 4a, see Methods)^24^. Restraint stress reduced the firing rates of VTA DA neurons (Supplementary Fig. 4a). In contrast to VTA MUA, the lag of peak phase-locking of VTA DA neural firing did not show a net directionality and did not change with restraint (Fig. 4b), suggesting that these neurons do not induce NAc stress oscillations. To test this hypothesis, we inhibited VTA DA neurons with archaerhodopsin and compared the power of NAc restraint-induced oscillations. Inhibiting VTA DA neurons did not significantly reduce NAc restraint-induced oscillations, making these neurons unlikely to drive stress-induced reward-seeking deficits (Supplementary Fig. 4c).

### VTA GABA activity during restraint is necessary for the low-frequency NAc oscillation and subsequent impaired reward-seeking

**Fig. 5:**
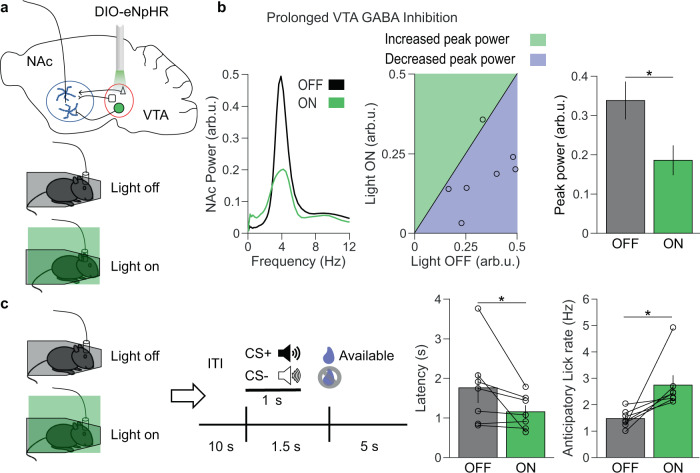
Prolonged VTA GABA inhibition during restraint decreases low-frequency NAc oscillations and rescues reward-seeking behavior. **a** Experimental design. **b** Left: example NAc LFP spectra during light off (black) and light on (green) sessions. Center: relationship between peak 4 Hz NAc power during light off and light on sessions, with a line of equality for reference. Right: summary of data from the center panel. Prolonged VTA GABA inhibition reduced peak 4 Hz NAc power (*P = 0.014 two-tailed paired *t* test, *n* = 7 mice). Data are mean ± s.e.m. **c** Left: experimental design. Right: Prolonged VTA GABA inhibition during restraint improved reward retrieval latency and anticipatory lick rate (*P = 0.031 and *P = 0.016, two-tailed signed-rank test, *n* = 7 mice). Data are mean ± s.e.m.

Although the majority of VTA neurons are dopaminergic (65%), the VTA also contains GABAergic neurons (25%)^25^, a subset of which project to the NAc^26^. We, therefore, injected Cre-dependent AAV-Arch into the VTA of Vgat-ires-Cre mice to optogenetically tag these GABA neurons (Fig. 4c)^24^. Restraint stress also reduced the firing rates of VTA GABA neurons (Supplementary Fig. 4b). However, the lag of peak phase-locking of VTA GABA neural firing was shifted to the past during restraint, indicating that VTA GABA neuron activity precedes the NAc restraint-induced oscillation, similar to the VTA MUA (Fig. 4d). Furthermore, inhibiting VTA GABA neurons reduced restraint-induced NAc oscillations (Supplementary Fig. 4d, e). We observed that Arch inhibition of VTA GABA neurons waned over the course of 1 minute, whereas halorhodopsin (eNpHR) robustly inhibited VTA GABA neurons for 30 mins (Supplementary Fig. 5a, b). Thus, to determine the role VTA GABA-mediated NAc, oscillations play in stress-induced reward-seeking deficits, we inhibited VTA GABA neurons throughout restraint stress using eNpHR. As expected, prolonged inhibition of VTA GABA neurons during restraint reduced low-frequency NAc oscillations (Fig. 5a, b), but inhibiting VTA GABA neurons in the familiar environment or with control virus had no impact on NAc oscillations (Supplementary Fig. 5c, 6a). Critically, inhibiting VTA GABA neurons during restraint reversed subsequent reward-seeking deficits, decreasing latency to lick for reward and increasing anticipatory licking (Fig. 5c and Supplementary Fig. 6b), suggesting that the activity of VTA GABA neurons during stress is necessary for subsequent disrupted reward-seeking.

### Rhythmic VTA GABA stimulation is sufficient to produce low-frequency NAc oscillations and impair reward-seeking

**Fig. 6:**
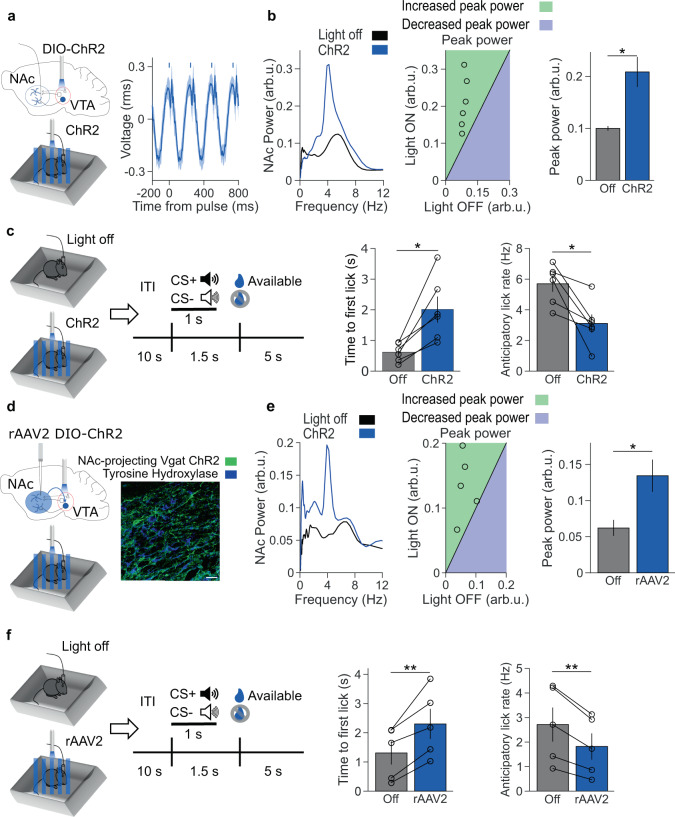
Activation of NAc-projecting VTA GABA neurons produces a low-frequency oscillation and impairs reward-seeking. **a** Left: experimental design. Right: event-related potential of NAc LFP. Bars indicate 10 ms laser pulse onset. **b** Left: representative NAc spectra with no stimulation (black) and rhythmic low-frequency stimulation (blue). Center: relationship between max 4 Hz NAc power during light off and stimulation, with a line of equality for reference. Right: summary of data from the center panel. ChR2 stimulation increased peak 4 Hz NAc power (*P = 0.031 two-tailed signed-rank test, *n* = 6 mice). Data are mean ± s.e.m. **c** Left: experimental design. Right: rhythmic ChR2 stimulation impaired subsequent reward retrieval latency and anticipatory lick rate (*P = 0.031 and *P = 0.031, two-tailed signed-rank test, *n* = 6 mice). Data are mean ± s.e.m. **d** Left: experimental design. Right: immunofluorescent image of retrograde ChR2 expression (green) in a Vgat-ires-Cre mouse VTA, with tyrosine hydroxylase counterstain (blue). The scale bar is 30 µm. Representative of five experiments. **e** Left: representative NAc spectra of no stimulation and rhythmic low-frequency stimulation. Center: relationship between max 4 Hz NAc power during light off and stimulation, with the line of equality for reference. Right: Summary of data from the center panel. Stimulating NAc-projecting VTA GABA neurons increased peak 4 Hz NAc power (*P = 0.040 two-tailed paired *t* test, *n* = 5 mice). Data are mean ± s.e.m. **f** Left: experimental design. Right: rhythmic VTA illumination impaired subsequent reward retrieval latency and anticipatory lick rate (**P = 0.0091 and **P = 0.0057 two-tailed paired *t* test, *n* = 5 mice). Data are mean ± s.e.m.

To assess if VTA GABA neural activity is sufficient to induce a low-frequency NAc oscillation, we injected Cre-dependent AAV carrying channelrhodopsin-2 (ChR2) into the VTA of Vgat-ires-Cre mice. Stimulating these VTA neurons at a low-frequency (4 Hz) drove the emergence of a low-frequency oscillation in the NAc (Fig. 6a, b, and Supplementary Fig. 6c). Next, we tested the impact of stimulating VTA GABA neurons on reward anticipation. Thirty minutes of low-frequency VTA GABA neuron stimulation in a familiar environment increased reward latency and decreased anticipatory licking during the subsequent reward-seeking session (Fig. 6c and Supplementary Fig. 6d). Stimulating VTA GABA neurons at 20 Hz did not produce an oscillation in the NAc, although it did result in modest reductions in reward-seeking (Supplementary Fig. 7a, b). Receiver operating characteristic (ROC) analysis confirmed that 4 Hz stimulation produced a more robust behavioral alteration than 20 Hz stimulation (Supplementary Fig. 7c). These results indicate that low-frequency VTA GABA stimulation suffices to induce low-frequency rhythmic NAc activity and to reduce subsequent reward-seeking, mimicking the effect of stress on NAc physiology and reward behavior.

However, it remained unclear whether direct GABAergic projections from VTA to NAc suffice to induce low-frequency oscillations and blunt reward-seeking. Previous studies reported that stimulating the terminals of NAc-projecting VTA GABA neurons does not impact reward-seeking^27–29^. However, we speculated that robust, rhythmic stimulation mimicking the activity seen during stress might be necessary to reduce reward anticipation. We, therefore, injected the Cre-dependent retrogradely transported virus rAAV2 carrying channelrhodopsin into the NAc of Vgat-ires-Cre mice. Stimulating the VTA cell bodies of NAc-projecting GABA neurons at low frequencies for 30 mins in a familiar environment-induced rhythmic NAc activity and blunted subsequent reward-seeking, with increased reward latency and decreased anticipatory licking, indicating that this small VTA GABAergic subpopulation can recapitulate both the physiologic signature of restraint stress and its impact on reward-seeking (Fig. 6d–f).

### Stress-induced blunted reward-seeking reflects a disruption in the VTA GABA, not DA, encoding of reward anticipation

**Fig. 7:**
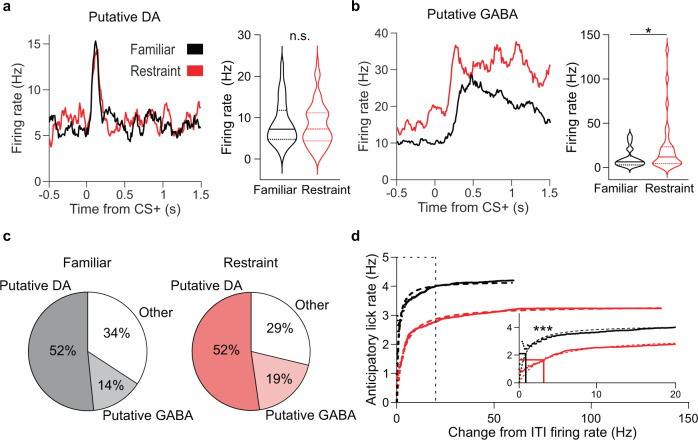
Restraint enhances VTA GABA activity but weakens its relationship with anticipatory behavior. **a** Left: representative CS+ evoked firing rates of putative DA neurons in non-stressed (black) and stressed (red) conditions. Right: CS+ evoked firing rates for putative DA neurons recorded in non-stressed and stressed conditions. Restraint did not affect the CS+ evoked firing rates of putative DA neurons (*P* = 0.64, two-tailed rank-sum test, *n*_Familiar_ = 80 neurons, *n*_Restraint_ = 80 neurons). Data plotted are median, upper and lower quartiles, and kernel density estimate. **b** The same as **a**, but for putative GABA neurons. Restraint enhanced CS+ evoked firing of putative GABA neurons (*P = 0.028, two-tailed rank-sum test, *n*_Familiar_ = 22 neurons, *n*_Restraint_ = 29 neurons). Data plotted are median, upper and lower quartiles, and kernel density estimate. **c** Distribution of putative DA and GABA neurons recorded during familiar environment and restraint stress conditions (total neurons *n*_Familiar_ = 155, *n*_Restraint_ = 153). **d** Relationship between cumulative change in firing rate of putative GABA neurons and average anticipatory lick rate for the familiar environment and restraint stress conditions. Dotted lines are nonlinear least-squares fitted functions. Inset: Same data graphed up to 20 Hz. Solid lines indicate EC50 value calculated for the fitted function. The EC50 value for the familiar environment fitted function was smaller than that of the restraint stress fitted function (***P < 0.0001 two-tailed ANCOVA).

The emergence of VTA GABA neurons as the key drivers of stress-induced reward circuit activity suggests a mechanism by which stress disrupts reward-seeking. The orchestrated activity of VTA DA and GABA neurons underlies reward processing. VTA DA neurons exhibit brief phasic activity in response to reward-predicting cues while VTA GABA neurons respond to reward-predicting cues with prolonged firing, resulting in characteristic firing patterns for each cell type (Fig. 7a–c). We reasoned that restraint stress might alter these firing patterns, causing decreased reward anticipation. After undergoing restraint, cue-evoked activity in putative DA neurons remained intact, but cue-evoked activity in putative GABA neurons was increased (Fig. 7a, b). Enhanced cue-evoked VTA GABA activity in mice with reduced motivation seemed counterintuitive, as these neurons are thought to subtract the expected value of reward in reward prediction calculations^6^. To investigate the relationship between putative VTA GABA activity and anticipatory behavior, we plotted the average anticipatory lick rate as a function of cumulatively increasing firing rates (Fig. 7d). We found that in unstressed mice, anticipatory lick rates increase as putative GABA firing rates increase, indicating that VTA GABA neuron activity reflects trial-to-trial fluctuations in motivation. However, following restraint stress, the maximum anticipatory lick rate and potency of GABA firing to produce anticipatory licking were both reduced (Fig. 7d). These data indicate that stress-induced low-frequency activity of VTA GABA neurons alters their normal function even after the stress has abated, disrupting the ability of these neurons to produce anticipatory behavior. Collectively, these data reveal a VTA-NAc circuit mechanism by which stress can cause reward-seeking deficits.

## Discussion

The convergence of stress and reward processing in the VTA-NAc circuit leads to the hypothesis that stress alters the capacity of the VTA-NAc circuit to appropriately respond to reward. However, previous efforts to link VTA-NAc DA circuit activity impacted by stress to reward-seeking deficits have yielded mixed results^8–10,30,31^. Here, we directly tested the role of VTA DA and GABA projections to the NAc during restraint and subsequent reward-seeking. We show that during acute restraint stress, VTA GABA neurons entrain rhythmic activity in the NAc, producing a 4 Hz NAc oscillation. In the aftermath of this stress, mice pursue rewards less avidly, with decreased anticipatory licking and prolonged latency to retrieve rewards. Concomitantly, reward-predicting cues evoke exaggerated VTA GABA neural responses. Remarkably, the strength of the NAc restraint oscillation predicts subsequent deficits in reward anticipation. Furthermore, we establish that VTA GABA activity is both necessary and sufficient for stress-induced reward-seeking deficits, as inhibiting VTA GABA neurons during stress improves reward-seeking, and mimicking stress-evoked activity in NAc-projecting VTA GABA neurons depresses reward pursuit in unstressed mice. Finally, we demonstrate that in unstressed mice, VTA GABA neural activity encodes motivation and that stress disrupts this relationship.

Notably, the acute stress in our study produced transient reward-seeking deficits, whereas the anhedonia observed in depression is long-lasting^4^. The protracted anhedonia in depression has been hypothesized to result from chronic stress^4^. Consistent with this, repeated stress exposure is a model of chronic stress that produces protracted reward-seeking deficits in mice^3,10^. Additional studies are necessary to determine the role of NAc-projecting VTA GABA neurons in the transition from chronic stress to lasting reward-seeking deficits.

VTA GABA neurons are well-situated to mediate the impact of stress on reward-seeking^32^. These neurons fire in response to aversive stimuli^5^ and play a crucial role in calculating reward prediction^6^. Moreover, they are increasingly recognized to play important roles in stress-mediated addiction behavior^33^. Interestingly, we found that VTA GABA neurons were inhibited during restraint stress, whereas previous studies have suggested these neurons are excited by aversive stimuli^5,28,34^. This discrepancy may arise from the modality or duration of our stressor. We began recording neural activity several seconds after the onset of stress, so it is possible that there is a transient increase in VTA GABA firing rate at the immediate onset of stress followed by inhibition as the stressor persists.

The subpopulation of VTA GABA neurons that project to the NAc may preferentially inhibit NAc cholinergic interneurons^35^. These cholinergic interneurons represent <1% of NAc neurons^36^, yet ramify broadly within the striatum^22^, inhibiting ~80% of NAc neurons^37^. As a result, low-frequency rhythmic VTA GABA neuron inhibition of NAc cholinergic neurons could underlie the widespread restraint-induced NAc LFP oscillation and single-unit firing modulation. Lending further support to this hypothesis, selectively silencing NAc cholinergic interneurons leads to decreased sucrose preference^38^.

We note that stimulating VTA GABA neurons at 20 Hz did not induce a NAc oscillation but did reduce reward-seeking, albeit modestly. A ROC analysis revealed that behavioral data can classify 4 Hz versus familiar sessions, but not 20 Hz versus familiar sessions. These findings suggest that while the activity of these neurons broadly modulates reward anticipation (and therefore even artificial stimuli have the potential to impact behavior), the observed 4 Hz oscillations that naturally occur in the context of stress represent the most-efficient activity pattern for blunting reward anticipation.

We find that rhythmically stimulating NAc-projecting VTA GABA neurons recapitulates the impact of stress on NAc physiology and reward anticipation. Interestingly, previous studies have reported decreased reward consumption during global VTA GABA, but not NAc-projecting VTA GABA neuron activation^27–29^. This difference might reflect stimulation parameters (continuous vs. rhythmic, axon terminal vs. retrograde cell body) or a difference in reward paradigms. Indeed, a recent report suggests that activating NAc-projecting VTA GABA neurons may selectively modulate aspects of reward anticipation^39^.

VTA neurons exhibit considerable heterogeneity^25,40^. Our study found that GABA, but not DA, neurons mediate stress-induced oscillations. Nonetheless, it is highly likely that subpopulations of VTA DA (and glutamate neurons) contribute to the influence of stress on reward processing. Recent work has identified specific populations of DA neurons that fire action potentials in response to aversive experiences^41^. Similarly, a recent paper reported that NAc-projecting VTA glutamatergic neurons carry negative valence information and mediate stress-induced behaviors^42^. There is also evidence that this neural population plays a role in reward processing^40^. Further study is needed to unravel how these VTA subpopulations interact to mediate the effects of stress on reward processing.

Our study reveals the cellular mediators of a restraint-induced VTA-NAc oscillation. Long-range oscillations form an important motif in neural circuit communication^43^, yet the cellular mediators of these oscillations remain obscure in many circuits. One well-characterized exception is theta generation in the medial septohippocampal circuit where hippocampal-projecting medial septal GABA neurons are pacemakers setting the frequency of theta oscillations, whereas cholinergic and glutamatergic neurons act as current generators modulating the amplitude of theta oscillations^44,45^. We show that long-range VTA GABA projections also mediate restraint-induced NAc oscillations, suggesting that these neurons may be particularly well-suited to promote long-range oscillations.

Notably, the magnitude of the oscillations we observed with optogenetic stimulation of VTA GABA projections was smaller than that of stress-induced oscillations, which suggests that light illumination and/or opsin expression incompletely engages the full population of VTA GABA neurons recruited by stress. In addition, VTA GABA projections to the NAc may govern the rhythm (as opposed to the amplitude) of the NAc oscillation, similar to medial septal GABA projections to the hippocampus^44^. In support of this idea, we found that 20 Hz stimulation of VTA GABA neurons did not produce a 4 Hz oscillation in the NAc, similar to the inability of high-frequency optogenetic stimulation of medial septal GABA projections to the hippocampus to drive theta oscillations^46^. Notably, 20 Hz VTA GABA stimulation did yield modest reward-seeking deficits, further supporting a dissociation between the amplitude of the 4 Hz oscillation and reward-seeking behavior during artificial stimulation. Although pharmacological inactivation of the VTA completely abolishes the 4 Hz NAc oscillation, other circuit elements, including excitatory inputs or local interneurons, may modulate the amplitude of 4 Hz NAc oscillations^44^. For example, there is evidence that vHPC projections to the NAc are activated by aversive stimuli and mediate stress susceptibility^15,47,48^. Future studies will determine how other inputs and local microcircuitry contribute to the 4 Hz NAc oscillation.

Although long-range oscillations are typically used to assay the activity of long-range circuits^43^, restraint-induced oscillations seem to reflect the degree to which stress-induced VTA GABA activity induces plastic changes in VTA-NAc circuitry that outlast the stressor and result in impaired reward anticipation. Indeed, although 4 Hz NAc oscillations correlate with the extent of reward-seeking deficits, the oscillations terminate at the end of stress, and we observe reward-seeking deficits after cessation of 4 Hz VTA GABA stimulation. Thus, restraint-induced NAc 4 Hz oscillations set into motion the neural activity underlying the expression of disrupted reward-seeking (elevated non-rhythmic cue-evoked VTA GABA firing). Plasticity affecting VTA GABA neurons has been implicated in the response to cocaine^49^ and stress^32^ and represents an attractive therapeutic target^33^. Future work will determine the precise nature of the plasticity that reduces reward-seeking after stress. Since the clinical anhedonia seen with mood disorders lasts for weeks^50^, it will also be crucial to determine whether the VTA-NAc GABAergic mechanism that causes acute stress-induced reductions of reward-seeking also underlies longer-lasting disruptions in reward processing. Although we found that the 4 Hz NAc oscillation ended after release from restraint and reward-seeking behaviors returned to normal the following day, chronic activation of this circuit may produce longer-lasting plasticity that produces sustained reward-seeking deficits. Such a mechanism might explain the difference between transient anhedonia following acute stress and long-lasting anhedonia following chronic stress.

The identification of the VTA neurons that bidirectionally modulate this restraint-induced oscillation is crucial for distinguishing it from other oscillations with similar frequency ranges. Recent work has identified respiration-driven oscillations, which have been proposed to originate in the olfactory bulbs and piriform cortex and to represent the true source of NAc oscillations^17,18^. Our data showing that the oscillation does not synchronize with respiration, but that it does rhythmically organize NAc single units, that VTA muscimol infusions abolish restraint-induced oscillations, and that stimulating NAc-projecting VTA GABA neurons reproduces the oscillation (and behavior) collectively suggest that during restraint NAc-projecting VTA GABA neurons induce a true oscillation in the NAc.

Stress has previously been reported to induce low-frequency oscillations in the prefrontal cortex^16,51^. Interestingly, we also found a prominent restraint-induced oscillation in the prefrontal cortex. As the oscillation was largest in NAc and prefrontal cortex neurons did not phase-lock to the PFC oscillation, in this study we focused on the NAc and its inputs from the VTA. We note, however, that a similar frequency oscillation, mediated by VTA DA neurons, has been shown to coordinate activity between the VTA and prefrontal cortex during working memory^52,53^. Moreover, both VTA DA and GABA neurons also project to the prefrontal cortex^54^. The functional significance of this circuit remains relatively unexplored, although restraint stress increases c-fos expression in prefrontal-projecting DA neurons^55^. Future studies should determine whether VTA input to the prefrontal cortex during stress also induces oscillations and/or influences reward processing.

In this study, we describe a stress-induced NAc oscillation that predicts the extent of subsequent deficits in reward anticipation, which is frequently disrupted in clinical depression^4^. We find that rhythmic VTA GABA neuron activity is both sufficient and necessary for this NAc oscillation, and that stress enhances cue-evoked VTA GABA activity while disrupting the relationship between VTA GABA activity and motivation. Our findings thus reveal a VTA GABA-NAc neural circuit that underlies the transition from acute stress to blunted reward-seeking. Future work will investigate whether this circuitry underlies the long-lasting anhedonia seen in depressive disorders.

## Methods

### Subjects

Four to six months old male and female Vgat-ires-Cre homozygous mice, Dat-ires-Cre heterozygous mice, C57BL/6J mice, and 129/SvEv mice (The Jackson Laboratory, stock numbers 028862, 006660, 000664, and 002448, respectively) were used as experimental subjects. Mice were housed at 25 °C and 30% humidity with a standard 12 h light–dark cycle and were given ad libitum access to food and water (when not being water restricted for the cued-reward task). All procedures were approved by New York State Psychiatric Institute Institutional Animal Care and Use Committee at Columbia University, in accordance with NIH guidelines.

### Surgical procedures

Mice were anesthetized with 1–3% vaporized isoflurane in oxygen (1 L/min) and placed in a stereotaxic apparatus. Cre mice were injected with Cre-inducible Archaerhodopsin (AAV5-EF1a-DIO-eArch3.0-EYFP, UNC vector core), Cre-inducible Halorhodopsin (AAV5-Ef1a-DIO-eNpHR3.0-EYFP, Addgene), Cre-inducible ChR2 (AAV5-Ef1a-DIO-hChR2(E123T/T159C)-EYFP, Addgene), or control EYFP virus (AAV5-EF1a-DIO-EYFP, UNC vector core) at two locations within the VTA bilaterally (±0.5 mm ML and −3.4 mm AP from bregma, −4.5 mm DV from brain surface; 0.5 µL virus per injection. For projection-specific experiments, mice were injected with retrogradely transported Cre-inducible ChR2 (rAAV2-EF1a-DIO-hChR2(H134R)-EYFP-WPRE-HGHpA, Addgene) at eight locations within the NAc bilaterally (±1.8 mm ML, 0.98 mm AP, −4.12 mm DV; ±0.9 mm ML, 1.5 mm AP, −4.0 mm DV; ±0.6 mm ML, 1.5 mm AP, −3.4 mm DV; ±1.0 mm ML, 1.0 mm AP, −3.4 mm DV; 0.4 µL virus per injection). For microdrive implantation experiments, animals underwent a second surgery to implant the microdrive ~4 weeks after initial viral injection. Animals were again anesthetized and placed in a stereotaxic apparatus. Craniotomies were made to allow for implantation of a bundle of 15 stereotrodes (13 micron tungsten wire, California Fine Wire) and a 200 micron optical fiber in the left VTA (−0.5 mm ML, −3.4 mm AP, −4.45 mm DV); a 200 micron optical fiber in the right VTA; an LFP wire (76 micron tungsten wire, California Fine Wire) in the NAc (–1.25 mm ML, 1.25 mm AP, −4.0 mm DV); a ground screw over the cerebellum; and a reference screw over the olfactory bulb. Electrodes were connected to a 32 channel electrode interface board (EIB-36 Narrow, Neuralynx), and the board and wires were fixed to an advanceable custom microdrive. Electrode placements were confirmed by passing a current through an electrode at each site (5 mA, 10 s) to generate an electrothermolytic lesion. Mice were anesthetized with ketamine/xylazine prior to generating the lesions and were then perfused. The brains were extracted, cryoprotected, sectioned, mounted, stained, and examined under a microscope to determine lesion placements and characterize eYFP, Arch, eNpHR, and ChR2 expression in VTA.

### Optogenetics

In all, 561 nm wavelength light (Opto Engine, LLC), 532 nm wavelength light (OEM laser), or 473 nm wavelength (LaserGlow Technologies) light was delivered at 10 mW via 200 mm diameter, 0.22 NA optical fibers. For Arch experiments, 532 nm light was delivered in 60 s ON/60 s OFF epochs as mice explored a familiar environment and while mice were restrained. For eNpHR experiments, 532 nm or 561 nm light was delivered constantly for 30 mins while mice were restrained. In addition, 532 nm or 561 nm light was delivered 1 s ON/10 s OFF as mice explored a familiar environment for optogenetic tagging. For ChR2 experiments, mice either received no illumination or 4 Hz stimulation (10 ms pulse width) while exploring a familiar environment.

### Immunohistochemistry

At the conclusion of experiments (6–8 weeks after viral injection), mice were first anesthetized with a ketamine (100 mg/kg) and xylazine (7 mg/kg) mixture then perfused with 4% paraformaldehyde (Thermo Fisher Scientific), and the brains were extracted and cryoprotected in 30% sucrose in 1× PBS until they sank. Sections of 40 µm were taken in a cryostat and stored in 1× PBS at 4 °C until they were used for immunohistochemistry experiments. The following antibodies and dilutions were used: chicken polyclonal anti-GFP (1:500, Abcam, ab13970), Cy3 donkey anti-sheep (1:200, Jackson ImmunoResearch, 713-165-147), sheep polyclonal anti-tyrosine hydroxylase (1:1000, Abcam, ab113), Cy2 donkey anti-chicken (1:200, Jackson ImmunoResearch 703-225-155).

### Cued-reward task

After 5–7 days of post-surgical recovery, the mice were water restricted to 90% of baseline body weight. Prior to experimental recordings, the mice underwent 3 days of habituation to the recording setup: an opto/electrical tether was connected to the head stage preamplifier while the mice explored a small, rectangular, wooden box (24.5 cm × 34 cm) in 200 lux lighting conditions in 15–20 min daily sessions.

Upon reaching their target weight, the mice were trained on a cued-reward task using a custom-built, Arduino-driven rectangular, wooden box in which tones (1 kHz or 5–10 kHz white noise; 1 s duration) produced by a piezo buzzer (Digi-Key) predicted water rewards (15 µL) delivered with a solenoid valve (The Lee Company). The mice were presented with 150 trials of pseudo-randomly interleaved CS+ (predicting reward) or CS− (non-reward predicting). CS+/CS− identity was counterbalanced across mice. After the CS+ ended, there was 0.5 s delay, followed by a 5 s period during which reward would be delivered upon detection of a lick at the reward port by an infrared photo interrupter (SparkFun). This reward retrieval period was followed by a 10 s intertrial interval. Mice were trained until they retrieved rewards on at least 70% of CS+ trials for two consecutive days. Mice reached criterion performance in 6.5 ± 0.9 training sessions (mean ± s.e.m.). The number of training sessions needed did not correlate with the 4 Hz NAc power or the impaired reward behaviors, indicating that variable learning rates did not produce these effects (Supplementary Fig. 2i, j). Lick detection at the reward port was used to measure the first lick during the reward availability period and the rate at which mice would lick during reward anticipation and reward delivery.

To measure the diagnostic ability of behavioral data to identify stimulation and non-stimulation sessions, we generated ROC curves from reward retrieval latency and anticipatory lick rate data. The area under the curve (AUC) values was calculated and bootstrapping with 10,000 iterations was used to estimate confidence intervals for AUC values.

### Restraint

Mice were placed in tapered plastic film restraint bags (Decapicone) punctured with breathing holes before entering 50 mL plastic conical tubes, modified with a slit to allow the head stage and recording tether to pass. Restraint occurred for 30 min, after which mice were release from conical tubes and restraint bags. Videos of restraint were analyzed to determine periods of struggling. Each mouse was exposed to restraint stress only once unless otherwise noted. The mice used to characterize restraint-induced physiology in Fig. 1 and Supplementary Fig. 1 had previously undergone a 5 min social interaction test not analyzed in this study.

### Tail suspension

Mice were suspended 40 cm above the floor by taping their tails to a horizontal bar^56^. Video and electrophysiological recordings were collected for 6 min, after which mice were released. Videos were analyzed for periods of struggling.

### Pharmacological inactivation

Guide cannulas (26 gauge; Plastics One) were bilaterally implanted into the VTA as described above. An LFP wire was glued to the side of the cannula so that it extended 1.5 mm past the cannula tip. LFP wires were also implanted in the NAc. After recovery from surgery, the mice were habituated to the familiar environment for 3 days. Saline or muscimol (Tocris Bioscience) dissolved in saline (8.8 mM concentration) was microinfused into the VTA while the mice were in the home cage by backloading into a 33-gauge infusion cannula and into polyethylene (PE 20) tubing connected to 1.0 µL Hamilton microsyringe. The infusion cannula protruded 1 mm beyond the guide cannula tip. An infusion volume of 0.13 µL was delivered using a Harvard 11 Plus syringe pump (Harvard Apparatus) at a rate of 0.13 μL/min and the infusion cannula was left in place for 5 mins post infusion. After a 20–30 min post infusion interval, neural activity was recorded as the mice were placed in the familiar environment and then the restraint tube.

### Neural recordings

A Digital Lynx SX system with Cheetah 5.6.3 acquisition software (Neuralynx, Bozeman, MT) was used to amplify, a band-pass filter (1–1000 Hz for LFPs and 600–6000 Hz for spikes), and digitize the electrode recordings at sampling rates of 2 and 32 kHz for LFPs and spikes, respectively. Single units were clustered based on the first two principal components (peak and energy) from each channel using KlustaKwik 1.6 (Ken Harris) and visualized in Spike Sort 3D 2.5.1.0 (Neuralynx). Clusters were then visually inspected and included or eliminated based on waveform appearance, interspike interval distribution, isolation distance, and L-ratio.

### Respiratory recordings

To monitor respirations simultaneously with neural recordings, a 7 mm-long stainless-steel cannula (23 gauge, Small Parts, Inc) was implanted in the nasal cavity of mice that were also implanted with NAc LFP wires. Under anesthesia, a rectangular window 2 mm long and 1 mm wide was cut into the left nasal bone with a generic rotary 0.5 mm diameter ball end burr bit starting 2.5 mm from the caudal edge of the bone until nasal epithelium was visible. A solution of lidocaine was applied to the visible epithelium. An ~1.5 mm rostrocaudal incision was made in the epithelium, Gelfoam (Pfizer) was applied to stem any bleeding, and the preparation left to rest for 5 mins. The cannula was inserted into the incision such that the lower tip rested 0.5–1 mm below the surface of the epithelium. The cannula was affixed to the skull with Vetbond (3 M) and dental cement. The cannula was capped between experimental recordings. During experiments, the cannula was connected via polyethylene tubing (801000, A-M Systems) to a pressure sensor (24PCEFJ6G, Honeywell) and homemade preamplifier circuit^57^. The signal from this circuit was digitized at 32 kHz using the Digital Lynx system.

### Power, coherence, phase-locking analysis, and directionality analysis

Raw LFP data were normalized by dividing by the root mean square of the voltage signal while the mice were in the familiar environment. The power spectra and coherence of the LFPs were calculated with MATLAB wavelet functions (*cwt* and *wcoherence*). Power is reported in arbitrary units relative to baseline root mean square voltage. Power and coherence were calculated on 1-minute segments of data and averaged across the entire experimental session. To remove mechanical artifacts and EMG noise, indices with voltage values three standard deviations from the mean were omitted when averaging power. To speed-filter data, indices corresponding to when the animal was moving 0–5 cm/s were included when averaging power. To capture the animal-specific frequency of the restraint-induced oscillation, we measured the peak restraint-induced signal within a 4 Hz range and compared it with the corresponding signal in the familiar environment.

For phase-locking analysis, we digitally band-pass filtered the raw LFPs using a zero-phase delay filter (K. Harris and G. Buzsaki). The phase was calculated using a Hilbert transform, and a corresponding phase was assigned to each spike. We limited our analysis to units that fired at least 100 times over the period analyzed. Functional directionality was calculated based on the pairwise phase consistency (PPC) of VTA multiunit spikes assigned based on the corresponding phases of the 4 Hz filtered NAc LFP. We calculated PPC of VTA spikes that were shifted in 2.5 ms steps ±80 ms to 4 Hz NAc signals. We compared the lags of the maximum PPC value to determine VTA-to-NAc directionality, where negative lags correspond to VTA-to-NAc directionality.

### Single-unit analyses

We classified NAc single units as pMSNs, pTANs, or pFSIs based on the valley-amplitude ratio of spike waveforms, the coefficient of variation of the interspike interval (ISI CV), and the baseline firing rate^19–21^. pMSNs were identified by a low (<10 Hz) baseline firing rate and low (<3) valley-amplitude ratio. pTANs were identified by a low baseline firing rate and a high (>3) valley-amplitude ratio. pFSIs were identified by a high (>10 Hz) firing rate and a low (<2) ISI CV.

We identified significantly light-modulated units by bootstrapping. Laser ON and laser OFF spikes were combined, binned, and randomly shuffled 30,000 times. We considered units light-responsive if the observed difference between laser OFF and laser ON firing rate for a unit was >95% of the firing rate differences from the shuffled data.

To determine the effects of restraint effects on firing rate, the average firing rate was compared between a 5-minute baseline period during which mice explored a familiar environment and restraint during the same recording session. For task-evoked single-unit activity, spikes were binned in 10 ms bins and averaged across trials per neuron. CS+ evoked single-unit activity was calculated as the average firing rate per neuron during CS+ presentation. Identification of putative VTA DA and VTA GABA neurons was adapted from Cohen et al.^5^. Neurons were first filtered based on whether they were significantly task-modulated by performing a one-way analysis of variance of firing during a 1 s baseline period, during the 1 s CS+ presentation, and during the 0.5 s delay period. Significantly task-modulated units were then classified as putative VTA DA neurons if they had significantly higher reward-evoked firing in the 0–0.15 s period after reward retrieval relative to pre-CS+ baseline firing rate, as well as no difference between pre-CS+ baseline firing rate and delay period firing rate. Significantly task-modulated units were classified as putative VTA GABA neurons if they exhibited a higher firing rate during the delay period than the pre-CS+ baseline firing rate.

To quantify the efficacy of putative GABA firing in increasing average anticipatory lick rate, individual trials were discretized into 0.1 Hz bins of the average firing rate increase from the ITI period to the period between CS+ onset and reward availability. Trials in which the firing rate decreased between these periods were excluded. We fit a least-squares nonlinear function to describe the average anticipatory lick rate as a function of cumulative trials where putative GABA firing increased up to an increasing ceiling value. EC50 values from these fits were compared with an analysis of covariance test.

### Quantification and statistical analysis

Statistical analysis was performed in Graphpad Prism 9.0.1 or MATLAB 2018a. Two-tailed tests were used throughout. Data were tested for normality to determine whether to use parametric or non-parametric tests. For behavioral experiments, Wilcoxon signed-rank tests or paired *t* tests were used to assess the effects of stress and optogenetic manipulations within animals. For between-animal comparisons, Wilcoxon rank-sum tests or two-sample *t* tests were used. For calculation of the efficacy of Arch and eNpHR on oscillation inhibition, Bonferroni-corrected one-sample *t* tests were used to compare each time period to 100%. For all analyses, the alpha level was 0.05. Error bars and shaded error bands represent the s.e.m.

### Reporting summary

Further information on research design is available in the Nature Research Reporting Summary linked to this article.

## Supplementary information

Supplementary information

Reporting Summary

## Data Availability

The custom-written code used to analyze data is available from the corresponding author upon reasonable request.

## Source data

Source Data

## References

[1] Berghorst LH, Bogdan R, Frank MJ, Pizzagalli DA (2013). Acute stress selectively reduces reward sensitivity. Front. Hum. Neurosci..

[2] Wanat MJ, Bonci A, Phillips PE (2013). CRF acts in the midbrain to attenuate accumbens dopamine release to rewards but not their predictors. Nat. Neurosci..

[3] Harris AZ (2018). A novel method for chronic social defeat stress in female mice. Neuropsychopharmacology.

[4] Pizzagalli DA (2014). Depression, stress, and anhedonia: toward a synthesis and integrated model. Annu Rev. Clin. Psychol..

[5] Cohen JY, Haesler S, Vong L, Lowell BB, Uchida N (2012). Neuron-type-specific signals for reward and punishment in the ventral tegmental area. Nature.

[6] Eshel N (2015). Arithmetic and local circuitry underlying dopamine prediction errors. Nature.

[7] Sharpe MJ (2017). Dopamine transients are sufficient and necessary for acquisition of model-based associations. Nat. Neurosci..

[8] Wise RAJNR (2008). Dopamine and reward: the anhedonia hypothesis 30 years on. Neurotox. Res..

[9] Chaudhury D (2013). Rapid regulation of depression-related behaviours by control of midbrain dopamine neurons. Nature.

[10] Tye KM (2013). Dopamine neurons modulate neural encoding and expression of depression-related behaviour. Nature.

[11] Buzsáki G, Anastassiou CA, Koch C (2012). The origin of extracellular fields and currents—EEG, ECoG, LFP and spikes. Nat. Rev. Neurosci..

[12] Harris AZ, Gordon JA (2015). Long-range neural synchrony in behavior. Annu. Rev. Neurosci..

[13] Russo SJ, Nestler EJ (2013). The brain reward circuitry in mood disorders. Nat. Rev. Neurosci..

[14] Koenigs M, Grafman J (2009). The functional neuroanatomy of depression: distinct roles for ventromedial and dorsolateral prefrontal cortex. Behav. Brain Res..

[15] Bagot RC (2015). Ventral hippocampal afferents to the nucleus accumbens regulate susceptibility to depression. Nat. Commun..

[16] Kumar S (2014). Prefrontal cortex reactivity underlies trait vulnerability to chronic social defeat stress. Nat. Commun..

[17] Carmichael JE, Gmaz JM, van der Meer MAA (2017). Gamma oscillations in the rat ventral striatum originate in the piriform cortex. J. Neurosci..

[18] Tort AB, Brankačk J, Draguhn AJTIN (2018). Respiration-entrained brain rhythms are global but often overlooked. Trends Neurosci..

[19] Yarom O, Cohen D (2011). Putative cholinergic interneurons in the ventral and dorsal regions of the striatum have distinct roles in a two choice alternative association task. Front Syst. Neurosci..

[20] Gage GJ, Stoetzner CR, Wiltschko AB, Berke JD (2010). Selective activation of striatal fast-spiking interneurons during choice execution. Neuron.

[21] Atallah HE, McCool AD, Howe MW, Graybiel AM (2014). Neurons in the ventral striatum exhibit cell-type-specific representations of outcome during learning. Neuron.

[22] Tepper JM, Bolam JP (2004). Functional diversity and specificity of neostriatal interneurons. Curr. Opin. Neurobiol..

[23] Likhtik, E., Stujenske, J. M., Topiwala, M. A., Harris, A. Z. & Gordon, J. A. Prefrontal entrainment of amygdala activity signals safety in learned fear and innate anxiety. *Nat. Neurosci*. **17**, 106–113 (2014). 10.1038/nn.3582 PMC4035371 24241397

[24] Abbas AI (2018). Somatostatin interneurons facilitate hippocampal-prefrontal synchrony and prefrontal spatial encoding. Neuron.

[25] Morales M, Margolis EB (2017). Ventral tegmental area: cellular heterogeneity, connectivity and behaviour. Nat. Rev. Neurosci..

[26] Van Bockstaele E, Pickel V (1995). GABA-containing neurons in the ventral tegmental area project to the nucleus accumbens in rat brain. Brain Res..

[27] Wakabayashi, K. T. et al. Chemogenetic activation of ventral tegmental area GABA neurons, but not mesoaccumbal GABA terminals, disrupts responding to reward-predictive cues. *Neuropsychopharmacology* **44**, 372–380 (2019). 10.1038/s41386-018-0097-6 PMC6300533 29875446

[28] Tan KR (2012). GABA neurons of the VTA drive conditioned place aversion. Neuron.

[29] van Zessen R, Phillips JL, Budygin EA, Stuber GD (2012). Activation of VTA GABA neurons disrupts reward consumption. Neuron.

[30] Olney JJ, Warlow SM, Naffziger EE, Berridge KC (2018). Current perspectives on incentive salience and applications to clinical disorders. Curr. Opin. Behav. Sci..

[31] Zhong, W., Li, Y., Feng, Q. & Luo, M. J. J. o. N. Learning and stress shape the reward response patterns of serotonin neurons. **37**, 8863–8875 (2017). 10.1523/JNEUROSCI.1181-17.2017 PMC6596795 28821671

[32] Polter AM, Kauer JA (2014). Stress and VTA synapses: implications for addiction and depression. Eur. J. Neurosci..

[33] Bouarab, C., Polter, A. M. & Thompson, B. J. F. i. N. C. VTA GABA neurons at the interface of stress and reward. **13**, 78 (2019). 10.3389/fncir.2019.00078 PMC6906177 31866835

[34] Zhou Z (2019). A VTA GABAergic neural circuit mediates visually evoked innate defensive responses. Neuron.

[35] Brown MT (2012). Ventral tegmental area GABA projections pause accumbal cholinergic interneurons to enhance associative learning. Nature.

[36] Rymar VV, Sasseville R, Luk KC, Sadikot AF (2004). Neurogenesis and stereological morphometry of calretinin‐immunoreactive GABAergic interneurons of the neostriatum. J. Comp. Neurol..

[37] Witten IB (2010). Cholinergic interneurons control local circuit activity and cocaine conditioning. science.

[38] Warner-Schmidt JL (2012). Cholinergic interneurons in the nucleus accumbens regulate depression-like behavior. Proc. Natl Acad. Sci..

[39] Wakabayashi, K. T. et al. Chemogenetic activation of mesoaccumbal gamma-aminobutyric acid projections selectively tunes responses to predictive cues when reward value is unexpectedly decreased. *Biol. Psychiatry* **89**, 366–375 (2020). 10.1016/j.biopsych.2020.08.017 PMC8570639 33168181

[40] Root DH, Estrin DJ, Morales M (2018). Aversion or salience signaling by ventral tegmental area glutamate neurons. iScience.

[41] de Jong JW (2019). A neural circuit mechanism for encoding aversive stimuli in the mesolimbic dopamine system. Neuron.

[42] Qi J (2016). VTA glutamatergic inputs to nucleus accumbens drive aversion by acting on GABAergic interneurons. Nat. Neurosci..

[43] Harris, A. Z. & Gordon, J. A. Long-range neural synchrony in behavior. *Annu. Rev. Neurosci.,*10.1146/annurev-neuro-071714-034111 (2015). 10.1146/annurev-neuro-071714-034111 PMC4497851 25897876

[44] Buzsáki GJN (2002). Theta oscillations hippocampus. Neuron.

[45] Vandecasteele, M. et al. Optogenetic activation of septal cholinergic neurons suppresses sharp wave ripples and enhances theta oscillations in the hippocampus. *Proc. Natl. Acad. Sci. USA* **111**, 13535–13540 (2014). 10.1073/pnas.1411233111 PMC4169920 25197052

[46] Bender F (2015). Theta oscillations regulate the speed of locomotion via a hippocampus to lateral septum pathway. Nat. Commun..

[47] Muir J (2020). Ventral hippocampal afferents to nucleus accumbens encode both latent vulnerability and stress-induced susceptibility. Biol. Psychiatry.

[48] Pignatelli, M. et al. Cooperative synaptic and intrinsic plasticity in a disynaptic limbic circuit drive stress-induced anhedonia and passive coping in mice. *Mol. Psychiatry*, 10.1038/s41380-020-0686-8 (2020). 10.1038/s41380-020-0686-8 PMC7735389 32161361

[49] Bocklisch C (2013). Cocaine disinhibits dopamine neurons by potentiation of GABA transmission in the ventral tegmental area. Science.

[50] American Psychiatric Association. *Diagnostic and statistical manual of mental disorders (DSM-5®)*. (American Psychiatric Pub, 2013).

[51] Karalis N (2016). 4-Hz oscillations synchronize prefrontal-amygdala circuits during fear behavior. Nat. Neurosci..

[52] Fujisawa S, Buzsaki G (2011). A 4 Hz oscillation adaptively synchronizes prefrontal, VTA, and hippocampal activities. Neuron.

[53] Duvarci S (2018). Impaired recruitment of dopamine neurons during working memory in mice with striatal D2 receptor overexpression. Nat. Commun..

[54] Carr, D. B. & Sesack, S. R. GABA-containing neurons in the rat ventral tegmental area project to the prefrontal cortex. *Synapse* **38**, 114–123 (2000). 10.1002/1098-2396(200011)38:2<114::AID-SYN2>3.0.CO;2-R 11018785

[55] Deutch AY (1991). Stress selectively increases fos protein in dopamine neurons innervating the prefrontal cortex. Cereb. Cortex.

[56] Cerniauskas I (2019). Chronic stress induces activity, synaptic, and transcriptional remodeling of the lateral habenula associated with deficits in motivated behaviors. Neuron.

[57] Shusterman R, Smear MC, Koulakov AA, Rinberg D (2011). Precise olfactory responses tile the sniff cycle. Nat. Neurosci..

