# Supplementary material for: Ventral tegmental area GABA neurons mediate stress-induced blunted reward-seeking in mice
Source: Nat Commun. 2021 Jun 10;12:3539. doi: 10.1038/s41467-021-23906-2 (PMC8192742; doi:10.1038/s41467-021-23906-2)
Supplement: Supplementary file 1 — Supplementary information [file 41467_2021_23906_MOESM1_ESM.pdf]

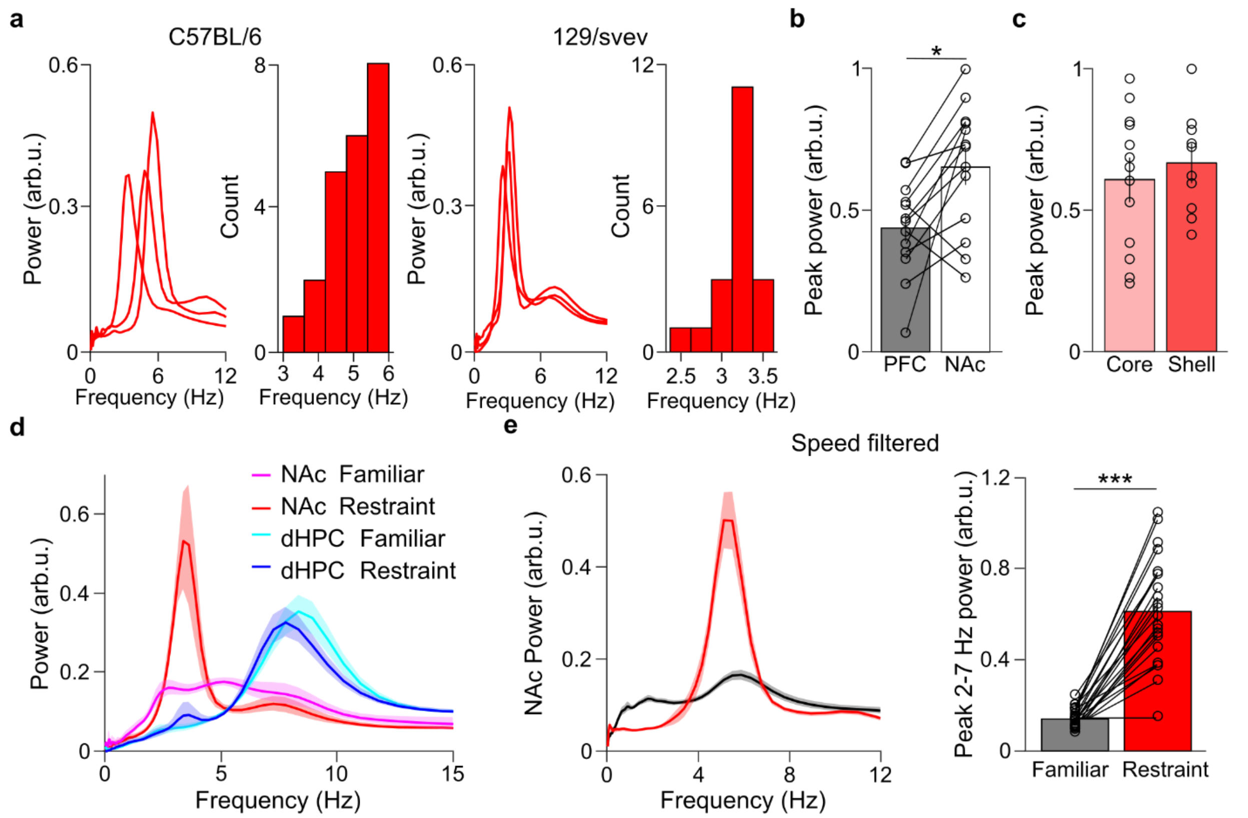

Supplementary Figure 1. A low-frequency restraint-induced oscillation occurs in the NAc LFP.

a, Left: Example NAc spectra from C57BL/6 strain mice and peak power frequency distribution of restraint-induced oscillation for C57BL/6 mice (n=22 mice). Right: Same as left, but for 129/svev strain mice (n=19 mice). **b,** Peak 2-7 Hz power during restraint was larger in the NAc than the PFC (*P=0.010, two-tailed Wilcoxon signed-rank test, n=13 mice). Data are mean ± s.e.m. **c**, Peak 2-7 Hz power was similar between NAc core and shell (P=0.44 two-tailed Wilcoxon rank-sum test, n_{core}=13 mice, n_{shell}=10 mice). Data are mean ± s.e.m. **d**, Averaged NAc and dHPC spectra (n_{NAc}=10 mice, n_{dHPC}=9 mice). Data are mean ± s.e.m. **e**, Left: Averaged NAc spectra during unstressed immobility and restraint stress. Right: Peak 2-7 Hz NAc power is larger during restraint

stress than unstressed immobility (***P<0.0001 two-tailed paired t-test, n=22 mice).

Data are mean ± s.e.m.

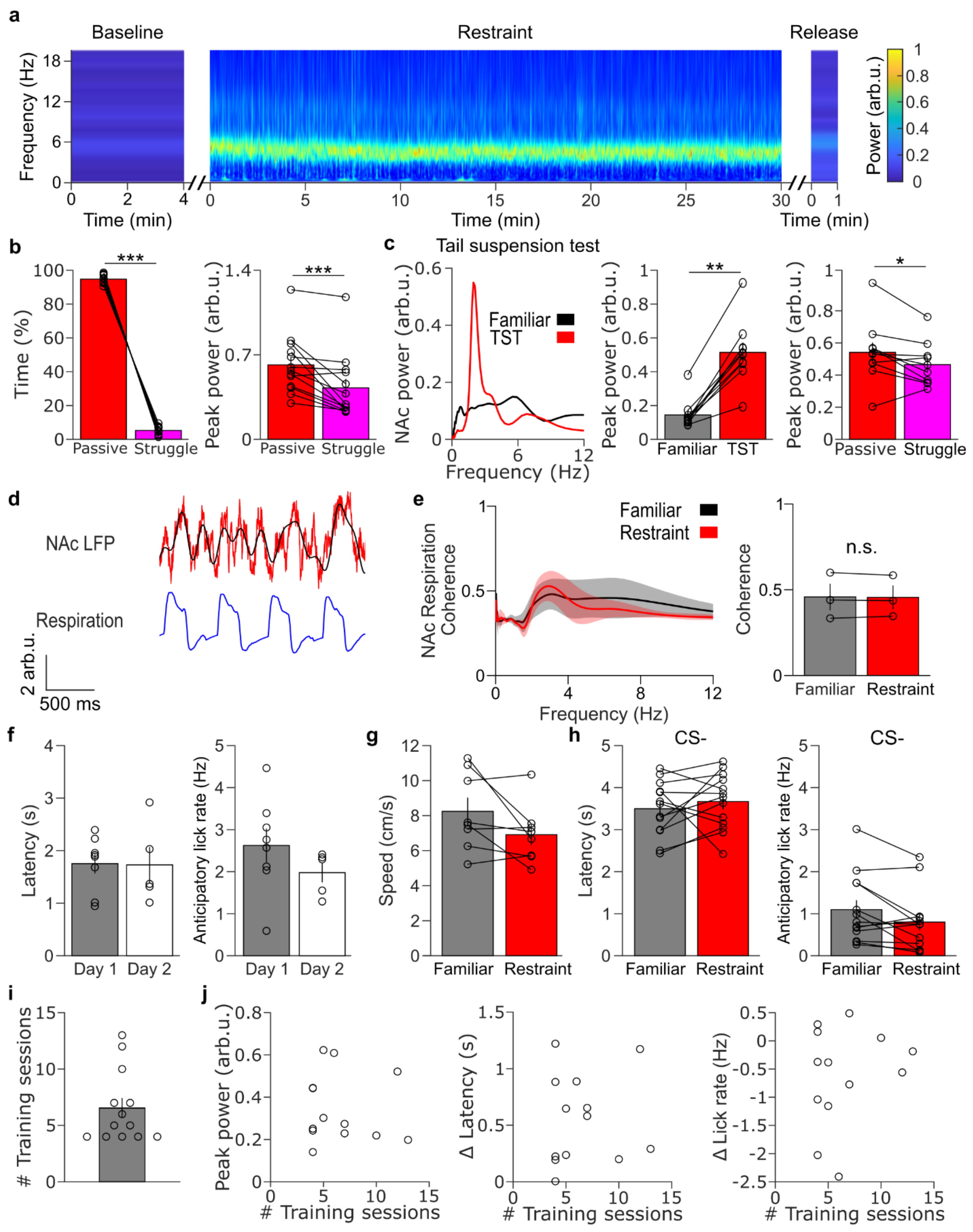

Supplementary Figure 2. The NAc restraint oscillation occurs with social stress and is not related to respiration.

a, Averaged spectrogram of NAc during pre-restraint baseline, restraint, and release from restraint (n=11 mice). **b**, Left: During restraint mice spent more time immobile than struggling (***P=0.00024 two-tailed Wilcoxon signed-rank test, n=13 mice). Right: Peak 2-7 Hz NAc power decreased during struggle events during restraint (***P=0.00073 two-tailed Wilcoxon signed-rank test, n=13 mice). Data are mean ± s.e.m. **c**, Left: Representative NAc spectra from familiar environment (black) and tail suspension (red). Center: Tail suspension increased peak 2-7 Hz NAc power (**P=0.0020 two-tailed Wilcoxon signed-rank test, n=10 mice). Right: Peak 2-7 Hz NAc power decreased during struggle events during tail suspension (*P=0.049 two-tailed Wilcoxon signed-rank test, n=10 mice). Data are mean ± s.e.m. **d**, Representative traces of the NAc LFP (red; 2-7 Hz filtered signal black overlay) and nasal cavity pressure measurements of respiration (blue) recorded from a mouse during restraint. **e**, Left: Coherence of NAc LFP and nasal cavity pressure. Right: Restraint does not significantly increase average LFP-Respiration coherence in the 2-7 Hz range (P=0.88, two-tailed paired t-test n=3 mice). Data are mean ± s.e.m. **f**, Restraint did not impair reward retrieval latency or anticipatory lick rate 24 hours later (P=0.95 and P=0.26, two-tailed two-sample t-test; n_{Day1}=8 mice, n_{Day2}=5 mice). Data are mean ± s.e.m. **g**, Restraint did not impair subsequent locomotor activity (P=0.18 two-tailed paired t-test, n=8 mice). Data are mean ± s.e.m. **h**, Restraint did not impair lick latency (P=0.43, two-tailed paired t-test, n=13 mice) or anticipatory lick rate (P=0.10, two-tailed Wilcoxon signed-rank test, n=13 mice) on CS- trials. Data are mean ± s.e.m. **i**, Number of training sessions needed to reach criterion performance in the cued-reward task. Data are mean ± s.e.m. **j**, The number of training sessions needed did not correlate with peak 2-7 Hz NAc power

during restraint or the restraint-induced changes in reward retrieval latency or anticipatory lick rate (P=0.80, P=0.78, and P=0.49, Pearson correlation, n=13 mice). Data are mean ± s.e.m.

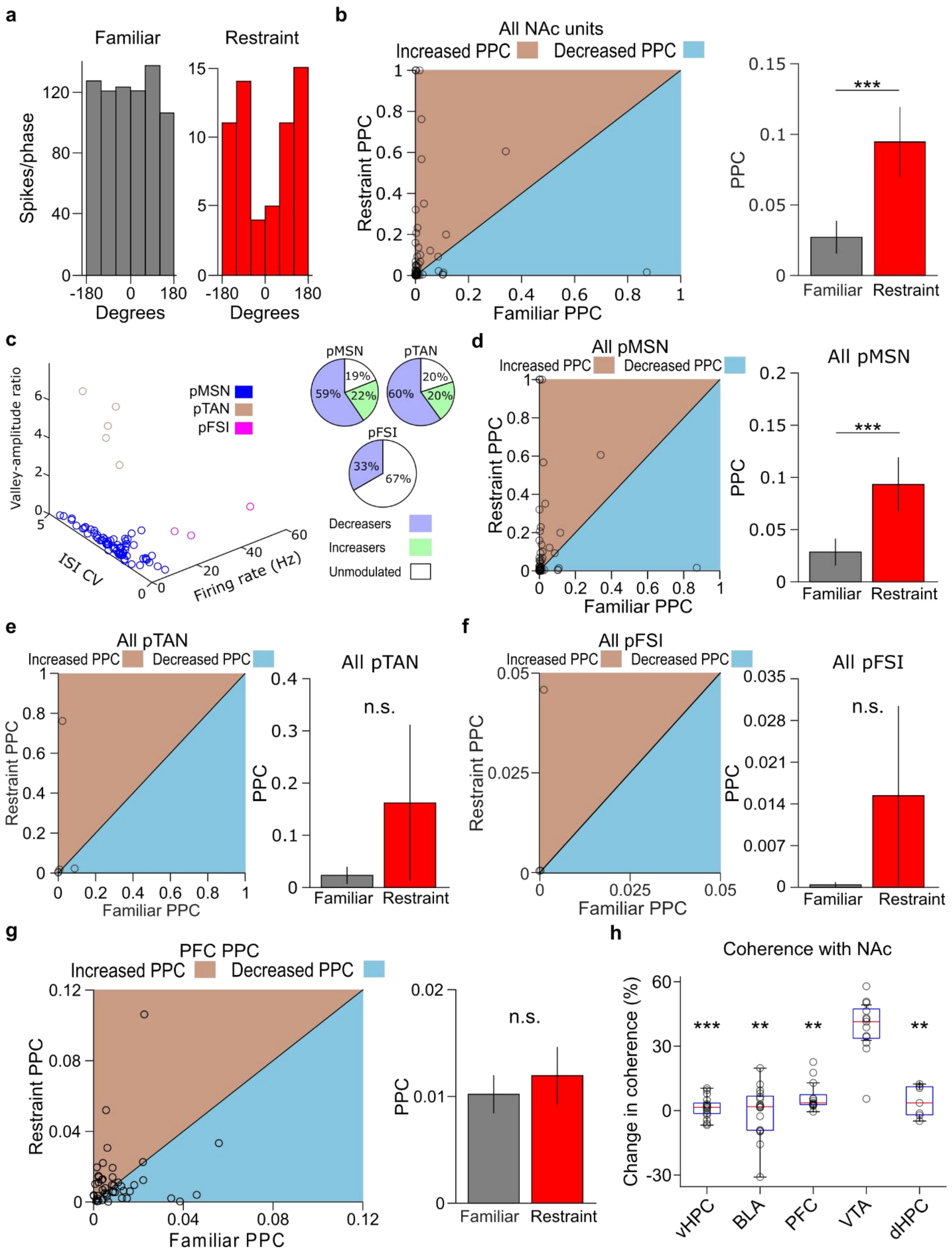

Supplementary Figure 3. Identification of the VTA as possible source of the NAc

restraint oscillation.

a, The spike-phase relationship for a restraint-inhibited NAc neuron. Activity was uniformly distributed across 2-7 Hz NAc LFP phase angles during familiar environment exploration, but phase-locked during restraint. **b**, Left: Relationship between the PPC of all NAc neurons during familiar environment exploration and restraint stress, with line of equality for reference. Right: Summary of data from left panel. Restraint increased phase-locking between NAc single units and the 2-7 Hz NAc LFP (***$P<0.0001$, two-tailed Wilcoxon signed-rank test, n=81 neurons). Data are mean ± s.e.m. **c**, Left: All recorded NAc units plotted by valley-amplitude ratio, interspike interval coefficient of variation (ISI CV), and baseline firing rate. Cells were clustered into putative medium spiny neuron (pMSN, blue), putative tonically active neurons (pTAN, brown), and putative fast-spiking interneurons (pFSI, magenta). Right: Distribution of pMSNs excited, inhibited, and unmodulated by restraint. **d**, Left: Relationship between the PPC of all pMSNs during familiar environment exploration and restraint stress, with line of equality for reference. Right: Summary of data from left panel. Restraint increased phase-locking of pMSNs to NAc 4 Hz (***$P<0.0001$ two-tailed Wilcoxon signed-rank test, n=73 neurons). Data are mean ± s.e.m. **e**, Left: Relationship between the PPC of all pTANs during familiar environment exploration and restraint stress, with line of equality for reference. Right: Summary of data from left panel. Restraint did not significantly affect phase-locking of pTANs to NAc 4 Hz ($P=0.44$ two-tailed Wilcoxon signed-rank test, n=5 neurons). Data are mean ± s.e.m. **f**, Left: Relationship between the PPC of all pFSIs during familiar environment exploration and restraint stress, with line of equality for reference. Right: Summary of data from left panel. Restraint did not

significantly affect phase-locking of pFSIs to NAc 4 Hz (P=0.25 two-tailed Wilcoxon signed-rank test, n=3 neurons). Data are mean ± s.e.m. **g**, Left: Relationship between the PPC of PFC neurons with the 2-7 Hz PFC LFP during familiar environment exploration and restraint stress, with line of equality for reference. Right: Summary of data from left panel. Restraint did not affect phase-locking between PFC neurons and the 2-7 Hz NAc LFP (P=0.50 two-tailed Wilcoxon signed-rank test, n=47 neurons). Data are mean ± s.e.m. **h**, Change in the average 2-7 Hz coherence of the NAc LFP with LFPs in the vHPC, BLA, PFC, VTA, and dHPC from familiar to restraint. The increase in VTA-NAc coherence exceeded that of all other regions (P<0.0001 Kruskal-Wallis, two-tailed post-hoc Tukey-Kramer comparisons ***P_{vHPC}<0.0001, **P_{BLA}=0.0077, **P_{PFC}=0.0063, **P_{dHPC}=0.0044, n_{vHPC}=17 mice, n_{BLA}=17 mice, n_{PFC}=13 mice, n_{VTA}=13 mice, n_{dHPC}=7 mice). Data are plotted with median, upper and lower quartiles, and 1.5x interquartile range.

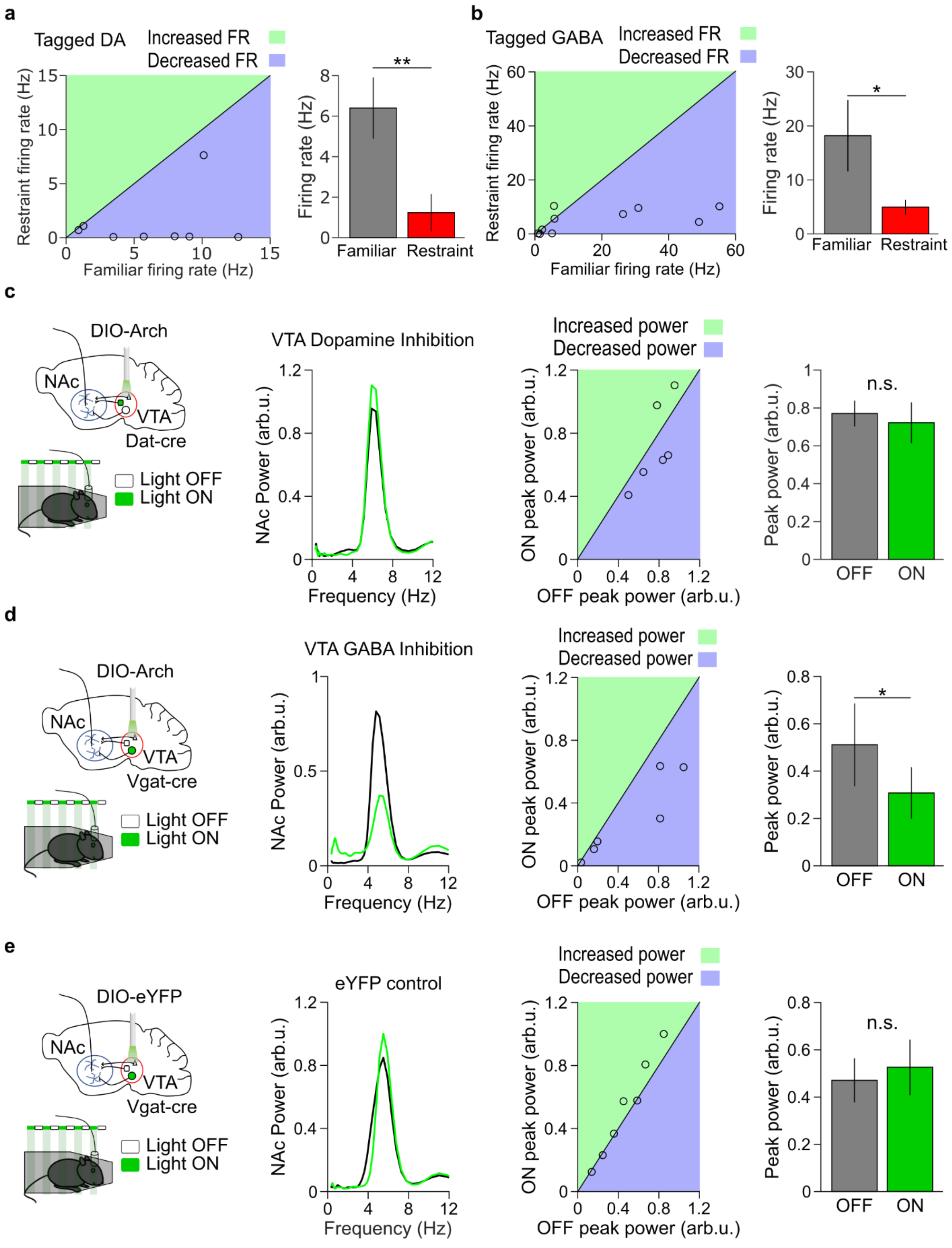

Supplementary Figure 4. VTA GABA inhibition reduces the stress-induced NAc

oscillation.

a, Left: Relationship between opto-tagged VTA DA neuron firing rates (FR) during familiar and restraint, with line of equality for reference. Right: Restraint inhibited VTA DA firing rate (**P=0.0078 two-tailed Wilcoxon sign-rank test, n=8 neurons). Data are mean ± s.e.m. **b**, The same as **a**, but for opto-tagged VTA GABA neurons. Restraint inhibited VTA GABA firing rate (*P=0.027 two-tailed Wilcoxon sign-rank test, n=10 neurons). Data are mean ± s.e.m. **c**, Far left: Experimental design. Left: Representative NAc spectra during light off (black) and on (green) epochs. Right: Relationship between peak 2-7 Hz NAc power during light off and on epochs, with line of equality for reference. Far right: Summary of data from right panel. Arch inhibition of VTA DA neurons did not affect peak 2-7 Hz NAc power (P=0.56, two-tailed Wilcoxon signed-rank test, n=6 mice). Data are mean ± s.e.m. **d,** Same as **c**, but for VTA GABA inhibition. Arch inhibition of VTA GABA neurons reduced peak 2-7 Hz NAc power (*P=0.031 two-tailed Wilcoxon signed-rank test, n=6 mice). Data are mean ± s.e.m. **e**, Same as **d**, but for eYFP controls. Illumination of the VTA did not affect peak 2-7 Hz NAc power (P=0.11 two-tailed paired t-test, n=7 mice). Data are mean ± s.e.m.

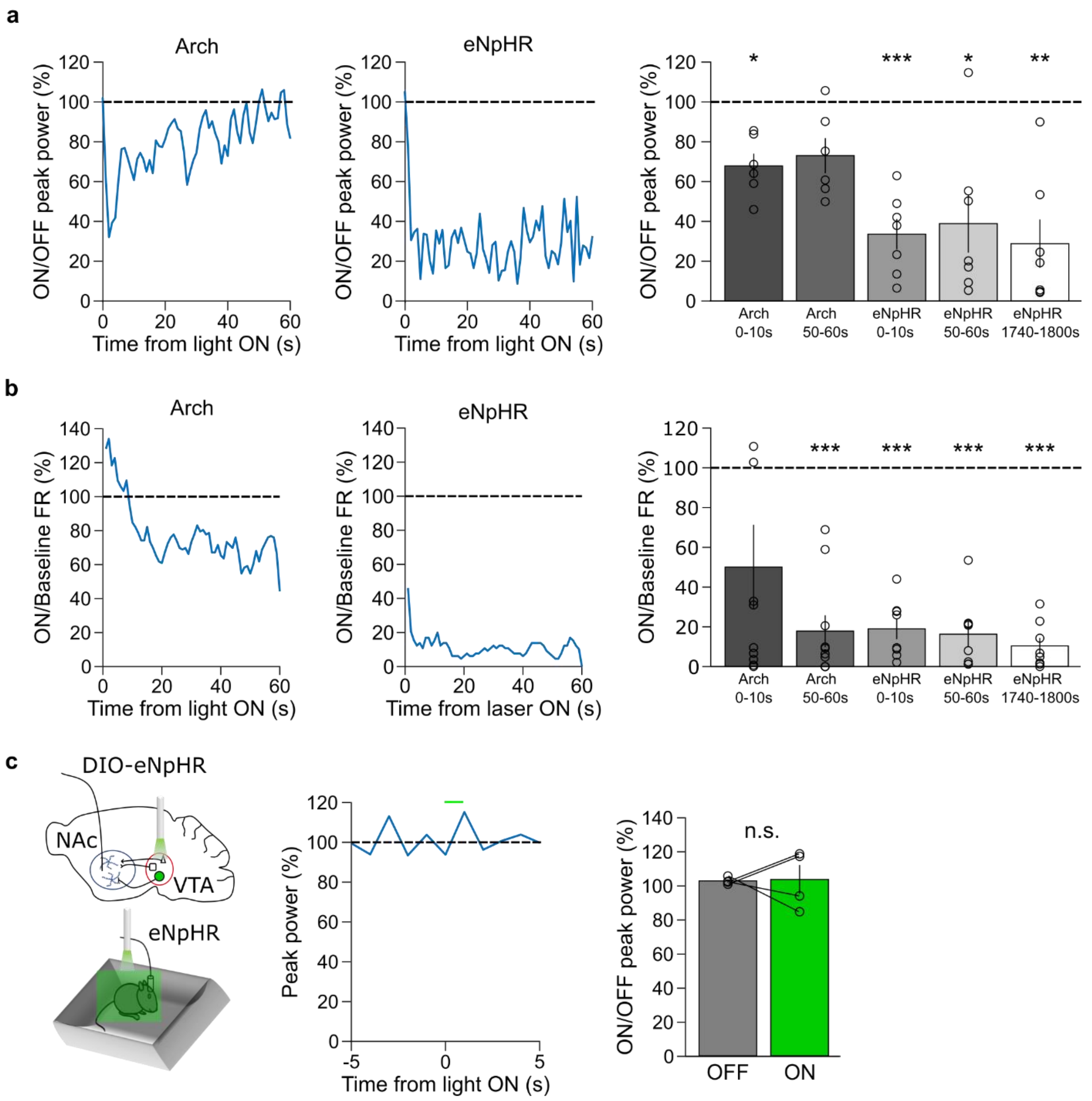

Supplementary Figure 5. Prolonged inhibition of the restraint oscillation with eNpHR.

a, Left: Example traces comparing efficacy of inhibiting 2-7 Hz NAc restraint oscillation with Arch and eNpHR VTA GABA inhibition. Right: Arch VTA GABA inhibition decreased the 2-7 Hz oscillation during the first 10 s of illumination (Bonferroni adjusted *P<0.05 one-sample t-test, n=6 mice) but not during the last 10 s of illumination

(Bonferroni adjusted $P>0.05$). By contrast, eNpHR VTA GABA inhibition decreased peak 2-7 Hz NAc power throughout restraint (Bonferroni adjusted $*P_{Arch0\text{-}10s}=0.017$, $P_{Arch50\text{-}60s}=0.14$, $***P_{eNpHR0\text{-}10s}=0.00063$, $*P_{eNpHR50\text{-}60s}=0.029$, $**P_{eNpHR1740\text{-}1800s}=0.0054$, two-tailed one-sample t-test, $n_{Arch}=6$ mice, $n_{eNpHR}=7$ mice). Data are mean ± s.e.m. **b**, The same as **a**, but for efficacy of inhibiting opto-tagged VTA GABA neuron firing rates (FR). Arch significantly reduced the firing rates of tagged GABA neurons 50-60 s after illumination onset (Bonferroni adjusted ***P<0.0001 two-tailed one-sample t-test, n=10 neurons), but not 0-10 s after illumination onset (Bonferroni adjusted P=0.22). eNpHR successfully inhibited tagged VTA GABA neurons throughout restraint (All Bonferroni adjusted ***P<0.0001 two-tailed one-sample t-test, n=8 neurons). Data are mean ± s.e.m. **c**, Left: Experimental design. Center: Example trace of effect of eNpHR inhibition on peak 2-7 Hz NAc power in familiar environment. Right: VTA GABA inhibition during familiar environment exploration has no effect on peak 2-7 Hz NAc power (P=0.93, two-tailed paired t-test, n=4 mice). Data are mean ± s.e.m.

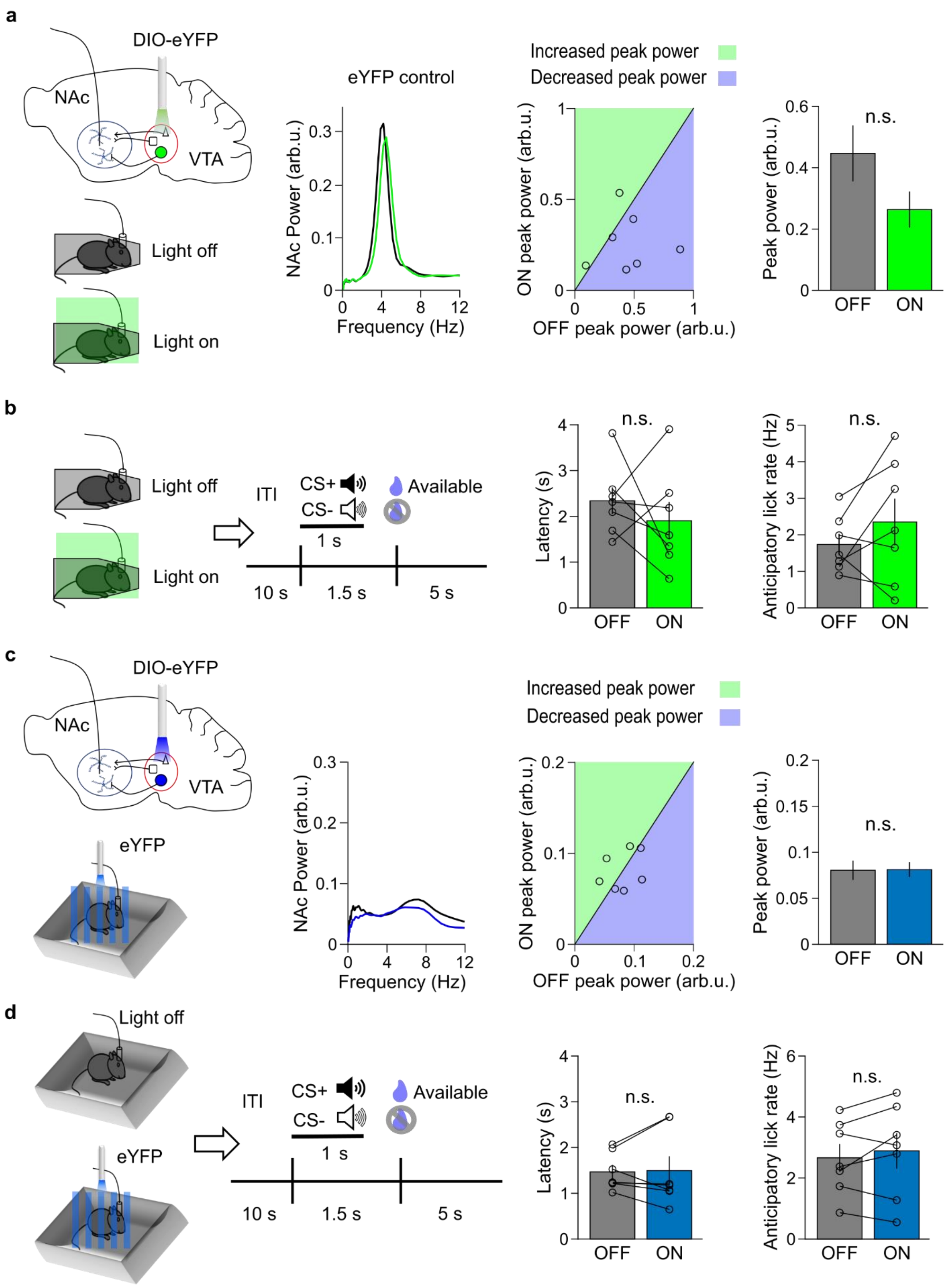

a
DIO-eYFP
NAc
VTA
Light off
Light on
eYFP control
NAc Power (arb.u.)
Frequency (Hz)
Increased peak power
Decreased peak power
ON peak power (arb.u.)
OFF peak power (arb.u.)
Peak power (arb.u.)
n.s.
OFF
ON
b
Light off
Light on
ITI
CS+
CS-
Available
1 s
10 s
1.5 s
5 s
Latency (s)
Anticipatory lick rate (Hz)
n.s.
OFF
ON
c
DIO-eYFP
NAc
VTA
eYFP
NAc Power (arb.u.)
Frequency (Hz)
Increased peak power
Decreased peak power
ON peak power (arb.u.)
OFF peak power (arb.u.)
Peak power (arb.u.)
n.s.
OFF
ON
d
Light off
eYFP
ITI
CS+
CS-
Available
1 s
10 s
1.5 s
5 s
Latency (s)
Anticipatory lick rate (Hz)
n.s.
OFF
ON

Supplementary Figure 6. eYFP controls for optogenetic experiments.

a, Far left: Experimental design. Left: Representative NAc spectra during restraint in light off (black) and light on (green) sessions. Right: Relationship between peak 2-7 Hz NAc power during light off and light on sessions, with line of equality for reference. Far right: Summary of data from right panel. Prolonged VTA illumination during restraint had no effect on peak 2-7 Hz NAc power (P=0.14 two-tailed paired t-test, n=7 mice). Data are mean ± s.e.m. **b,** Left: Experimental design. Right: Prolonged VTA illumination during restraint had no effect on reward retrieval latency or anticipatory lick rate (P=0.45 and P=0.27, two-tailed paired t-test, n=7 mice). Data are mean ± s.e.m. **c**, Far left: Experimental design. Left: Representative NAc spectra from familiar environment with no illumination (black) and rhythmic low-frequency illumination (blue). Right: Relationship between max 2-7 Hz NAc power during light off and rhythmic light illumination, with line of equality for reference. Far right: Summary of data from right panel. Rhythmic light illumination did not affect peak 2-7 Hz NAc power (P=0.95 two-tailed paired t-test, n=7 mice). Data are mean ± s.e.m. **d**, Left: Experimental design. Right: Rhythmic VTA illumination did not affect subsequent reward retrieval latency (P=0.94, two-tailed signed-rank test) or anticipatory lick rate (P=0.37, two-tailed paired t-test, n=7 mice). Data are mean ± s.e.m.

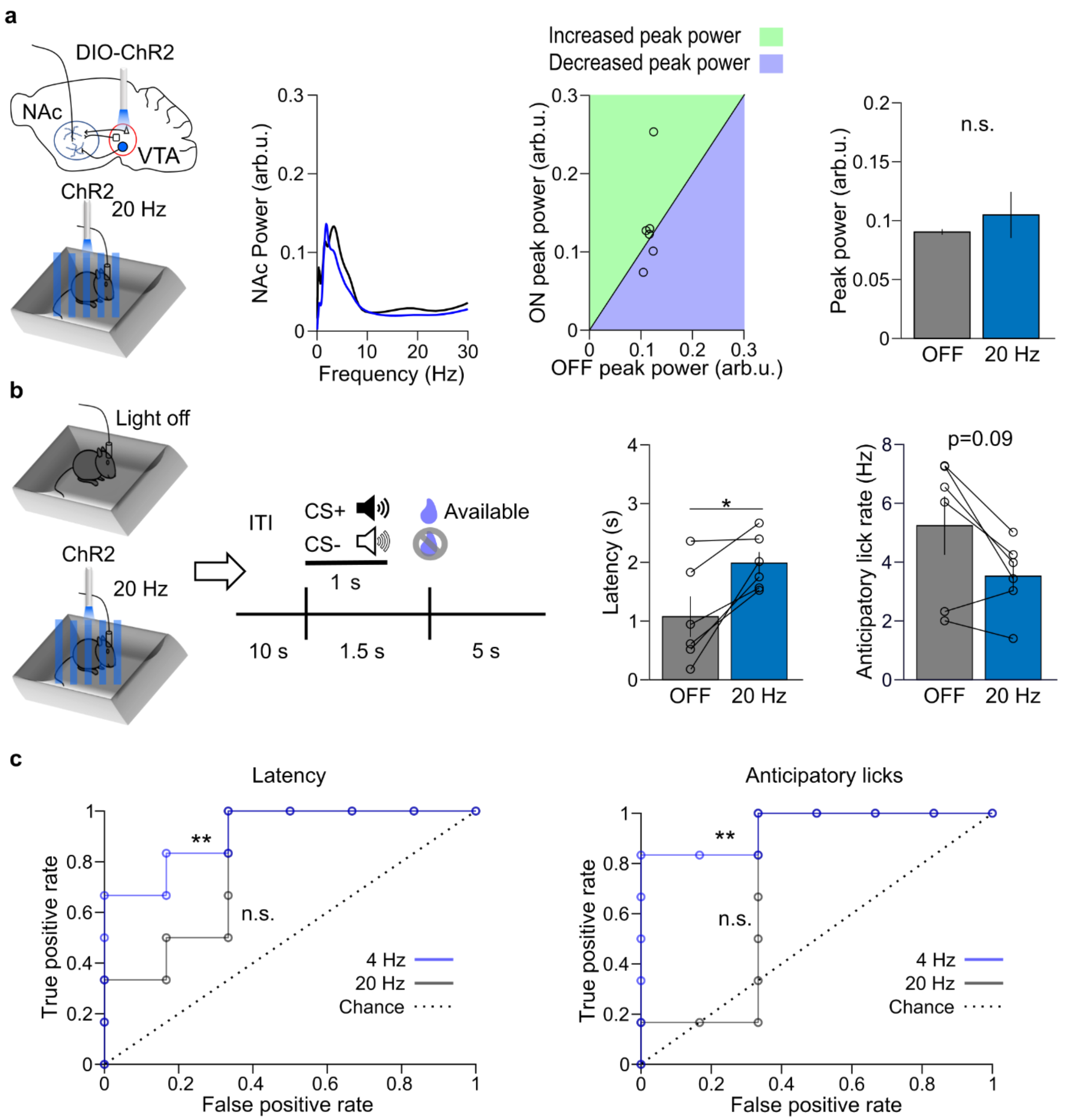

Supplementary Figure 7. Restraint disrupts the correlation between VTA GABA activity and reward anticipation.

a, Far left: Experimental design. Left: Representative NAc spectra from familiar environment with no stimulation (black) and rhythmic high-frequency stimulation (20 Hz, blue). Right: Relationship between peak 2-7 Hz NAc power during light off and rhythmic

light illumination, with line of equality for reference. Far right: Summary of data from right panel. 20 Hz stimulation did not affect peak 2-7 Hz NAc power (P=0.84 two-tailed signed-rank test, n=6 mice). Data are mean ± s.e.m. **b**, Left: Experimental design. Right: Rhythmic high-frequency VTA GABA stimulation resulted in an increase in reward retrieval latency (*P=0.014 two-tailed paired t-test, n=6 mice) and a non-significant decrease in anticipatory lick rate (P=0.094 two-tailed Wilcoxon signed-rank test, n=6 mice). Data are mean ± s.e.m. **c**, ROC curves describing diagnostic accuracy of behavioral data. Left: Reward retrieval latency data identified stimulation sessions better than chance with 4 Hz data (**AUC=0.917, 99% CI [0.583, 1.00]) but not better than chance with 20 Hz data (AUC=0.722, 95% CI [0.469,1]). Right: Anticipatory lick rate data identified stimulation sessions better than chance with 4 Hz data (**AUC=0.944, 99% CI [0.643, 1.00]) but not better than chance with 20 Hz data (AUC=0.694, 95% CI [0.333,1.00]).

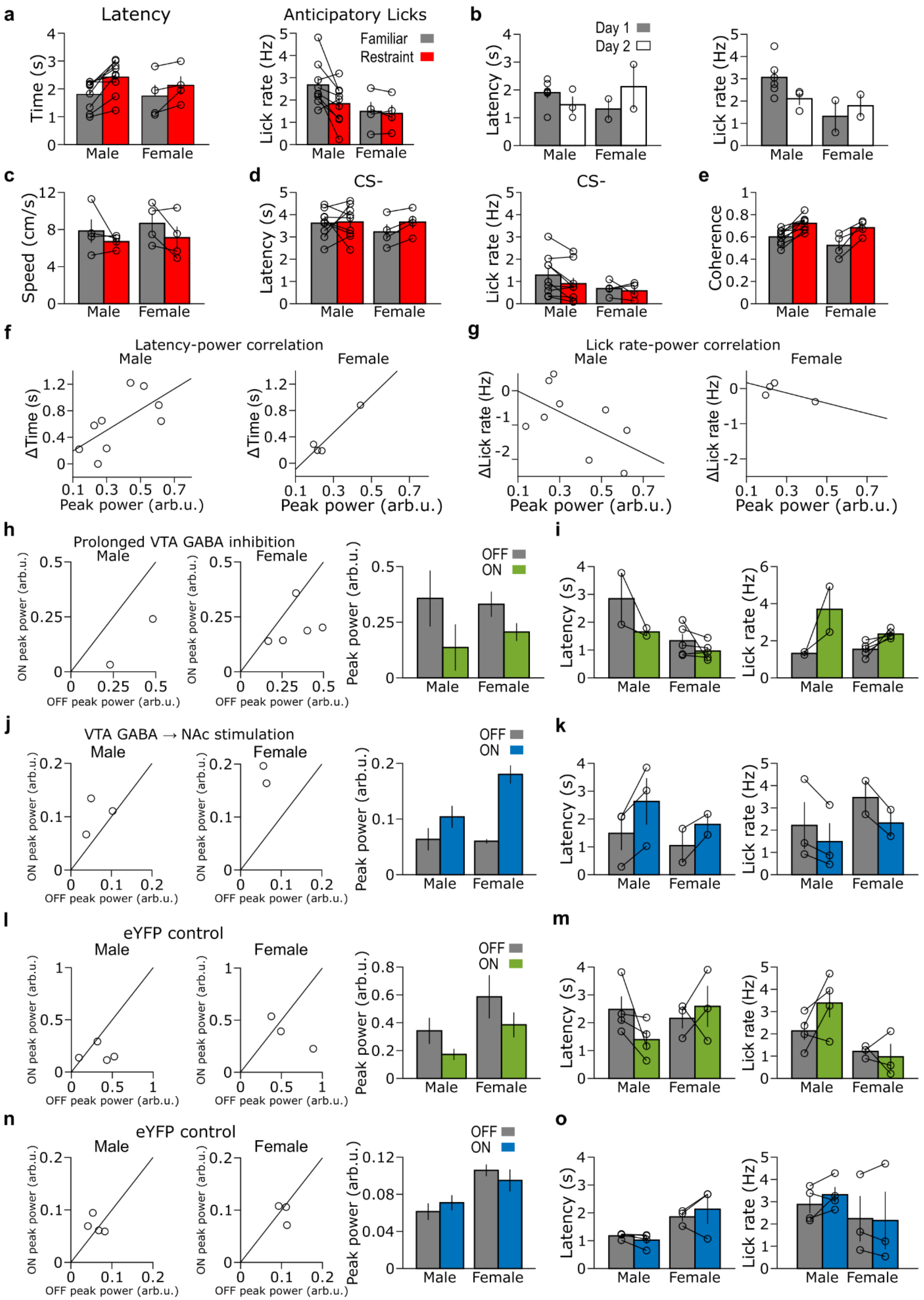

a
Latency
Time (s)
Male
Female
Anticipatory Licks
Familiar
Restraint
Lick rate (Hz)
b
Day 1
Day 2
Latency (s)
Lick rate (Hz)
c
Speed (cm/s)
d
CS-
Latency (s)
Lick rate (Hz)
e
Coherence
f
Latency-power correlation
ΔTime (s)
Peak power (arb.u.)
g
Lick rate-power correlation
ΔLick rate (Hz)
h
Prolonged VTA GABA inhibition
ON peak power (arb.u.)
OFF peak power (arb.u.)
OFF
ON
Peak power (arb.u.)
i
j
VTA GABA → NAc stimulation
k
l
eYFP control
m
n
o

Supplementary Figure 8. Data disaggregated by sex.

a, Left: Data from Figure 1d, disaggregated by sex. No effect of sex ($P>0.05$ 2-way repeated measures ANOVA). Right: Data from Figure 1d, disaggregated by sex. No effect of sex ($P>0.05$ 2-way repeated measures ANOVA). **b**, Data from Supplementary Figure 2f, disaggregated by sex. No effect of sex ($P>0.05$ 2-way repeated measures ANOVA). **c**, Data from Supplementary Figure 2g, disaggregated by sex. No effect of sex ($P>0.05$ 2-way repeated measures ANOVA). **d**, Data from Supplementary Figure 2h, disaggregated by sex. No effect of sex (both $P>0.05$ 2-way repeated measures ANOVA). **e**, Data from Figure 3a, disaggregated by sex. No effect of sex ($P>0.05$ 2-way repeated measures ANOVA). **f**, Data from Figure 1f, disaggregated by sex. No effect of sex ($P>0.05$ 2-way repeated measures ANOVA). **g**, Data from Figure 1g, disaggregated by sex. No effect of sex ($P>0.05$ 2-way repeated measures ANOVA). **h**, Left: Data from Figure 5b, disaggregated by sex. Right: Summary of data from left panel. No effect of sex ($P>0.05$ 2-way repeated measures ANOVA). **i**, Data from Figure 5c, disaggregated by sex. There was a main effect of sex on reward retrieval latency ($P<0.05$), but no significant sex*inhibition interaction ($P>0.05$, 2-way repeated measured ANOVA). There was no effect of sex on anticipatory lick rate ($P>0.05$ 2-way repeated measures ANOVA). **j**, Left: Data from Figure 6e, disaggregated by sex. Right: Summary of data from left panel. No effect of sex ($P>0.05$ 2-way repeated measures ANOVA). k, Data from Figure 6f, disaggregated by sex. No effect of sex (Both $P>0.05$ 2-way repeated measures ANOVA). **l**, Left: Data from Supplementary Figure 6a, disaggregated by sex. Right: Summary of data from left panel. There was a main effect of sex on peak NAc power ($P<0.05$), but no sex*laser interaction ($P>0.05$ 2-way repeated measures

ANOVA). **m**, Data from Supplementary Figure 6b, disaggregated by sex. There was no effect of sex on reward retrieval latency ($P>0.05$ 2-way repeated measures ANOVA). There was a main effect of sex on anticipatory lick rate ($P<0.05$), but no sex*laser interaction ($P>0.05$ 2-way repeated measures ANOVA). **n**, Left: Data from Supplementary Figure 6c, disaggregated by sex. Right: Summary of data from left panel. There was a main effect of sex on peak NAc power ($P<0.05$), but no sex*laser interaction ($P>0.05$ 2-way repeated measures ANOVA). **o**, Data from Supplementary Figure 6d, disaggregated by sex. There was a main effect of sex on reward retrieval latency ($P<0.05$), but no significant sex*inhibition interaction ($P>0.05$, 2-way repeated measured ANOVA). There was no effect of sex on anticipatory lick rate ($P>0.05$ 2-way repeated measures ANOVA). Data are mean ± s.e.m.
