# Supplementary material for: Ventral tegmental area GABA neurons mediate stress-induced blunted reward-seeking in mice
Source: Nat Commun. 2021 Jun 10;12:3539. doi: 10.1038/s41467-021-23906-2 (PMC8192742; doi:10.1038/s41467-021-23906-2)
Supplement: Supplementary file 2 — Reporting Summary [file 41467_2021_23906_MOESM2_ESM.pdf]

nature research

Corresponding author(s): Alexander Z. Harris

Last updated by author(s): May 12, 2021

Reporting Summary

Nature Research wishes to improve the reproducibility of the work that we publish. This form provides structure for consistency and transparency in reporting. For further information on Nature Research policies, see our Editorial Policies and the Editorial Policy Checklist.

Statistics

For all statistical analyses, confirm that the following items are present in the figure legend, table legend, main text, or Methods section.

n/a	Confirmed	
☐	☒	The exact sample size (*n*) for each experimental group/condition, given as a discrete number and unit of measurement
☐	☒	A statement on whether measurements were taken from distinct samples or whether the same sample was measured repeatedly
☐	☒	The statistical test(s) used AND whether they are one- or two-sided<br>*Only common tests should be described solely by name; describe more complex techniques in the Methods section.*
☐	☒	A description of all covariates tested
☐	☒	A description of any assumptions or corrections, such as tests of normality and adjustment for multiple comparisons
☐	☒	A full description of the statistical parameters including central tendency (e.g. means) or other basic estimates (e.g. regression coefficient) AND variation (e.g. standard deviation) or associated estimates of uncertainty (e.g. confidence intervals)
☐	☒	For null hypothesis testing, the test statistic (e.g. *F*, *t*, *r*) with confidence intervals, effect sizes, degrees of freedom and *P* value noted<br>*Give P values as exact values whenever suitable.*
☒	☐	For Bayesian analysis, information on the choice of priors and Markov chain Monte Carlo settings
☒	☐	For hierarchical and complex designs, identification of the appropriate level for tests and full reporting of outcomes
☒	☐	Estimates of effect sizes (e.g. Cohen's *d*, Pearson's *r*), indicating how they were calculated

Our web collection on statistics for biologists contains articles on many of the points above.

Software and code

Policy information about availability of computer code

Data collection	Neuralynx Cheetah Version 5.6.3
Data analysis	SpikeSort 3D 2.5.1.0, KlustaKwik 1.6, MATLAB R2018a, GraphPad Prism 9.0.1

For manuscripts utilizing custom algorithms or software that are central to the research but not yet described in published literature, software must be made available to editors and reviewers. We strongly encourage code deposition in a community repository (e.g. GitHub). See the Nature Research guidelines for submitting code & software for further information.

Data

Policy information about availability of data

All manuscripts must include a data availability statement. This statement should provide the following information, where applicable:

- Accession codes, unique identifiers, or web links for publicly available datasets
- A list of figures that have associated raw data
- A description of any restrictions on data availability

Source data are provided with this paper.

Field-specific reporting

Life sciences study design

All studies must disclose on these points even when the disclosure is negative.

Sample size	Power analysis (alpha: 5%, power: 80%) showed a sample size of 6 is sufficient to observe an effect of stress on behavior. As a result, we reduced the number of mice after our initial large cohorts.
Data exclusions	Single and multi-unit data from animals with inaccurate stereotrode wire implantations were excluded from phase-locking and cue-evoked firing experiments.
Replication	Behavioral findings were replicated with 2-3 cohorts and physiological findings were replicated with 3-6 cohorts.
Randomization	All mice experienced the stressor or optogenetic manipulation in a randomized order.
Blinding	Investigators were not blinded to group allocation during data collection due to the nature of the manipulations performed. Analyses were conducted blind to experimental condition.

Reporting for specific materials, systems and methods

We require information from authors about some types of materials, experimental systems and methods used in many studies. Here, indicate whether each material, system or method listed is relevant to your study. If you are not sure if a list item applies to your research, read the appropriate section before selecting a response.

Materials & experimental systems

n/a	Involved in the study	
☐	☒	Antibodies
☒	☐	Eukaryotic cell lines
☒	☐	Palaeontology and archaeology
☐	☒	Animals and other organisms
☒	☐	Human research participants
☒	☐	Clinical data
☒	☐	Dual use research of concern

Methods

n/a	Involved in the study	
☒	☐	ChIP-seq
☒	☐	Flow cytometry
☒	☐	MRI-based neuroimaging

Antibodies

Antibodies used	Chicken polyclonal anti-GFP (1:500, Abcam, ab13970), Cy3 donkey anti-sheep (1:200, JacksonImmunoResearch, 713-165-147), sheep polyclonal anti-TH (1:1000, Abcam, ab113), Cy2 donkey anti-chicken (1:200, Jackson ImmunoResearch 703-225-155).
Validation	Validation statements of commercial antibodies are available from manufacturers:<br>Chicken polyclonal anti-GFP (https://www.abcam.com/gfp-antibody-ab13970.pdf)<br>Cy3 donkey anti-sheep (https://www.jacksonimmuno.com/catalog/products/713-165-147)<br>sheep polyclonal anti-TH (https://www.abcam.com/tyrosine-hydroxylase-antibody-ab113.pdf)<br>Cy2 donkey anti-chicken (https://www.jacksonimmuno.com/catalog/products/703-225-155)

Animals and other organisms

Policy information about studies involving animals; ARRIVE guidelines recommended for reporting animal research

Laboratory animals	4-6-month-old male and female Vgat-ires-Cre homozygous mice, Dat-ires-Cre heterozygous mice, C57BL/6J mice, and 129/svev mice (The Jackson Laboratory, stock numbers 028862, 006660, 0006664, and 002448 respectively) were used as experimental subjects and housed in a facility kept at 25 °C and 30% humidity.
Wild animals	This study did not involve wild animals.
Field-collected samples	This study did not involve field-collected samples.
Ethics oversight	All procedures were carried out in accordance with the NIH Guidelines and approved by Columbia University and the New York State Psychiatric Institute Institutional Animal Care and Use Committees.

Note that full information on the approval of the study protocol must also be provided in the manuscript.
